# Assessing good environmental status through mesozooplankton biodiversity: a step forward

**DOI:** 10.1093/plankt/fbac067

**Published:** 2022-11-28

**Authors:** I Theodorou, S Zervoudaki, I Varkitzi, G Tsirtsis

**Affiliations:** DEPARTMENT OF BIOLOGY, NATIONAL AND KAPODISTRIAN UNIVERSITY OF ATHENS, ZOGRAFOU 15772, ATHENS, GREECE; INSTITUTE OF OCEANOGRAPHY, HELLENIC CENTRE FOR MARINE RESEARCH (HCMR), ANAVYSSOS 19013, ATTICA, GREECE; INSTITUTE OF OCEANOGRAPHY, HELLENIC CENTRE FOR MARINE RESEARCH (HCMR), ANAVYSSOS 19013, ATTICA, GREECE; DEPARTMENT OF MARINE SCIENCES, UNIVERSITY OF THE AEGEAN, UNIVERSITY HILL, 81100, MYTILENE, GREECE

**Keywords:** zooplankton, diversity indices, marine strategy framework directive, marine water quality, Saronikos Gulf, Aegean Sea, Eastern Mediterranean

## Abstract

We developed a zooplankton-based water-quality evaluating method using indices of alpha diversity. Two key objectives were set: (i) the comparison of two—different quality—samples from different areas, and the verification of their differentiation, based on mesozooplankton biodiversity indices; and (ii) the development of a methodology, which was able to assess the quality of new marine water samples. Our analysis was based on a 24-year-long *in situ* dataset (1987–2010) of 139 samples in which 86 mesozooplankton taxa were identified. High-diversity and high evenness values were reported in the case of the “good” status sample, while low diversity, low evenness and high dominance values occurred at the lower quality one. A linear discriminant analysis (LDA) was conducted that discriminated the tested samples at 100%. This LDA was then used to evaluate samples of unknown quality. Finally, 90% of them were classified with a probability of correct classification (posterior probability) >95%. The present study proves that mesozooplankton diversity indices can discriminate different levels of anthropogenic impacts. In this sense, it can be used as a reliable indicator for environmental assessment in the pelagic habitats of the Mediterranean Sea.

## INTRODUCTION

The EU Marine Strategy Framework Directive (MSFD; 2008/56/EC) is the first environmental directive dealing with the marine environment on an ecosystem approach, aiming to achieve Good Environmental Status (GES) for European seawater (Borja *et al.,* 2010). Each EU member state must perform regular assessments for eleven descriptors related to a state or a pressure or both, to assess seawater quality and monitor changes in environmental status (European Union, 2008). Through a number of “criteria” for assessing GES, the Directive ends up with the description of several indicators, defined as evaluation and decision tools, allowing a situation or trend to be measured (European Union, 2010). According to the MSFD, indicators must focus on significant biological components of the ecosystem, ranging from taxonomic groups to the whole ecosystem (Borja *et al.,* 2013; Berg *et al.,* 2015).

Zooplankton—functionally and phylogenetically diverse organisms that mainly drift with the water masses or slowly swim vertically—is a key group among the biological components listed in Table I of the Annex III of the MSFD (Cochrane *et al.,* 2010). Zooplankton research traditionally focuses on mesozooplankton (0.2–20 mm), due to its pivotal role in the marine ecosystem and in particular its contribution to the biological pump (Steinberg and Landry, 2017). Zooplankton has a great potential as an “observer” of “environmental changes and pressures” (Legendre and Rivkin, 2005; Richardson, 2008; Beaugrand *et al.,* 2010). It is an important element of the ecosystem which—apart from its role in deep ocean carbon export (Longhurst *et al.,* 1990; Steinberg *et al.,* 2002; Hannides *et al.,* 2009; Robinson *et al.,* 2010) and nutrient recycling in the upper layer (Siokou-Frangou *et al.,* 2010; Zervoudaki *et al.,* 2020)—links primary productivity with higher trophic levels (Palomera *et al.,* 2007; Bacha and Amara, 2009; Borme *et al.,* 2009). Moreover, unlike organisms under fishing pressure, zooplankton is not commercially exploitable in Europe (Zervoudaki *et al.,* 2020). Therefore, fluctuations in its abundance and diversity are solely due to environmental changes and pressures. Finally, due to its global presence, water quality indicators based on zooplankton can be tested and widely used in different ocean regions.

Zooplankton has been used for water quality assessment in previous studies (Arfi *et al.,* 1981; Siokou-Frangou and Papathanassiou, 1991; Uriarte and Villate, 2005; Kršinić *et al.,* 2007; Gorokhova *et al.,* 2013; Varkitzi *et al.,* 2018; Magliozzi *et al.,* 2021). In cases, the total or relative abundance or biomass of zooplankton, copepods and specific taxa or a combination of them (e.g. HELCOM Core Indicator of Biodiversity Mean Size and Total Stock; Gorokhova *et al.,* 2013) has been used to develop quality indices (Varkitzi *et al.,* 2018). There are currently five available—zooplankton based—regional state indicators, (pre)operational to defining GES, under MSFD (Magliozzi *et al.,* 2021). So far, zooplankton biomass and distributional range of zooplankton are the most popular indices under the MSFD concept (Varkitzi *et al.,* 2018). Other relevant studies—outside of the MSFD concept—mainly result in the identification of particular species, being more sensitive to reflect human pressures (e.g. Arfi *et al.,* 1981; Siokou-Frangou and Papathanassiou, 1991; Uriarte and Villate, 2005; Kršinić *et al.,* 2007).

Few attempts have been made to use ecological indices, expressing diversity, evenness and dominance of zooplankton communities, for seawater quality assessment. Species richness has been used by Beaugrand (2005), Beaugrand *et al.* (2010) and HELCOM (2012) in the form of the ratio of species observed and species number registered in an area. Simpson’s and Shannon’s diversity, and Piélou’s evenness indices have been also used (Beaugrand *et al.,* 2010; Aare *et al.,* 2014; Serranito *et al.,* 2016). In the Mediterranean Sea, few studies have attempted to develop a biodiversity-based index, using time series of zooplankton data (Serranito *et al.,* 2016; Villarino *et al.,* 2020). None of the five available zooplankton-based state indicators is designed, or has defined thresholds, for the Mediterranean Sea and its subregions (Magliozzi *et al.,* 2021).

**Table I:** Periods, number of samples, geographical coordinates and depth of sampling stations

Station	Periods of sampling	Years	Number of samples	Latitude, N	Longitude, E	Depth
S2	P-01 and P-02	1995–2010	24	38° 00.00	23° 27.18	35
S7	P-01 and P-02	1995–2010	31	37° 55.42	23° 35.45	75
S11	P-0, P-01 and P-02	1987–2010	84	37° 52.36	23° 38.30	82

The use of alpha diversity indices for water quality assessment has some important benefits. Being internationally accepted and used, they meet the requirements of widespread or even global use. They have been acknowledged as possible state indicators (ICG-COBAM, 2013; Aare *et al.,* 2014). Especially changes of biodiversity indices can be used for Habitat distribution (indicators 1.4.1, 1.4.2) and Habitat condition (indicator 1.6.1) (European Union, 2010). Those changes are mainly sensitive to multiple human-induced pressures such as “nutrients,” “selective extraction” and “climate change,” and to a lesser extent to “physical loss,” “physical damage,” “hydrological change,” “contaminants” and “non-indigenous species” (ICG-COBAM, 2013). Moreover, HELCOM (2012) reported that “Zooplankton species diversity” (an index using species richness) is directly impacted by “climatic changes” in thermal regime, pH and salinity, introduction of “synthetic compounds” and “invasive species.” All the above impose that zooplankton-relevant ecological indices seem to be appropriate as state indicators, reflecting multiple environmental pressures. However, appropriate diversity indices should be chosen based on certain criteria (discrimination between sites, dependence on sample size, diversity component being measured, wider usage and understanding of the index) (Varkitzi *et al.,* 2018). Given the multidimensional nature of biodiversity, a combination of existing indices should be also examined.

Measuring impacts on biodiversity only through species richness or a single diversity index—although popular enough (Johnston and Roberts, 2009)—has been characterized as “relatively crude”(Gray *et al.,* 1990) and“dubious”(Hurlbert, 1971). This type of indices would better be accompanied by metrics indicative of species evenness, or dominance, since tolerant species are expected to clearly dominate anthropogenically contaminated marine habitats (Pastorok and Bilyard, 1985; Hillebrand *et al.,* 2007). In other words, marine regions suffering of anthropogenic pollution frequently demonstrate reduced diversity, either due to reduced species richness, increased dominance of competitive ones or a combination of the above (Johnston and Roberts, 2009).

In the present work, we assess the efficiency of alpha diversity indices to express mesozooplankton diversity responses along different water qualities. The considered indices express all aspects of diversity including species richness, evenness and dominance. Two key objectives were set: (i) screening of a large number of indices for their sensitivity to discriminate mesozooplankton diversity changes between two tested conditions according to WFD/MSFD criteria, one characteristic of good quality and another of non-good water quality, and (ii) the development of a statistical methodology based on such sensitive indices, able to set limits for the evaluation of samples of unknown water quality. Our study is based on two hypotheses: (i) species richness is decreasing when moving from “good” to “non-good” environmental conditions, resulting in lower diversity and evenness, and (ii) some species are able to tolerate bad environmental conditions, have competitive advantage and develop high biomass, therefore increasing dominance and decreasing evenness (Thompson and Shin, 1983; Simboura *et al.,* 1995; Je *et al.,* 2004; Johnston and Roberts, 2009).

## METHOD

### Outline of the method

To test, evaluate and combine several alpha diversity indices for the development of a mesozooplankton-based index for seawater quality assessment, several steps were taken. Firstly, two sites were chosen, namely station and period of “good” and “non-good” status—based on previous studies on the selected area (Simboura and Zenetos, 2002; Simboura *et al.,* 2005, 2014; Zenetos *et al.,* 2005; Siokou-Frangou *et al.,* 2009; Pavlidou *et al.,* 2014). Then a screening of 27 ecological indices was performed, aiming to identify the most sensitive to discriminate the two conditions. Sensitive indices were then combined in a principal component analysis (PCA) to identify general trends of changes in alpha diversity. Finally, a small number of selected indices fulfilling the necessary assumptions (normal distribution and homoskedasticity) were used in a linear discriminant analysis (LDA) to develop a zooplankton-based quality index.

### Case study

**Fig. 1:**
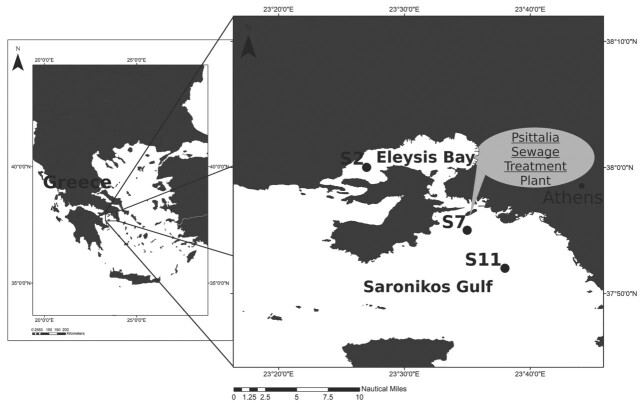
Map of the study area in Saronikos Gulf in Greece, indicating the sampling stations S2, S7 and S11 and the location of the Psittalia sewage treatment plant.

Samples from three coastal stations were used in the current study located in Saronikos Gulf (Aegean Sea, Greece) and sampled from 1987 to 2010 (Fig. 1). All samples were collected, using the same methodology throughout the years, with vertical tows of 200 μm mesh size net, from bottom of each station until the surface. The volume (m^3^) of filtered water (V = A × ΔL) was estimated considering the area of the net mouth (A) and the difference between the winch readings (L_i_ − L_f_). The first station (S2) is in Elefsis Bay and the rest (S7 and S11) in the inner part of the Gulf. Saronikos Gulf is in the oligotrophic Southern Aegean Sea (Eastern Mediterranean) and its water quality is affected by the sewage effluents of Athens metropolitan area (about 4 million inhabitants) (Siokou-Frangou *et al.,* 2009). Untreated sewage was discharged into the surface waters of Elefsis Bay and inner Saronikos Gulf until 1994 (Siokou-Frangou *et al.,* 2009). At this point, the operation of a first-stage treatment plant started in the Psittalia island, and the sewage was discharged into inner Saronikos Gulf at a depth of 63 m. The secondary stage of the Psittalia sewage treatment plant was functional by the end of 2004 (Siokou-Frangou *et al.,* 2009). Consequently, the period of study (1987–2010) was divided into three separate periods determined by the three operational phases of the Psittalia sewage treatment plant, coded as P-0 (1987–1994), P-01 (1995–2004) and P-02 (2005–2010) (Table I). A total of 139 mesozooplankton samples were used, in which 86 taxa (copepods and cladocerans) were identified at species level (or in a few cases at group of species). Zooplankton taxonomic identification and counting were carried out in the laboratory, using a dissecting microscope. Taxa abundance was expressed as number of individuals per cubic meter. Taxa abundance values were integrated—when necessary—to express abundance for combined depth layers. Data processing was performed on an annual basis, as well as separately for the warm (May–October) and the cold season (November–April).

### Selection of tested samples representative of “good” and “non-good” status

Two types of conditions were selected: one representative of “good” status (GES) including “high” and “good” environmental conditions and the other of “non-good” status (non-GES), representing “moderate,” “poor” and “bad” environmental conditions based on previous studies, on the use of Water Framework Directive (WFD; European Union, 2000) or MSFD criteria. According to those studies (data collected within periods P-0 and P-01), ecological quality of the inner Saronikos Gulf, based on the analysis of phytobenthic and macrozoobenthic communities, was ameliorated moving away from the sewage outfall (Siokou-Frangou *et al.,* 2009), and varied from “poor” at station S7 to “good” at station S11 (Simboura and Zenetos, 2002; Simboura *et al.,* 2005; Zenetos *et al.,* 2005). During the period P-02 (2005–2010), station S11 was characterized as of “good status” or oligotrophic in some cases, indicating a significant improvement in its ecological status (Pavlidou *et al.,* 2014; Simboura *et al.,* 2014). On the other hand, station S7 remained mesotrophic and S2 of “moderate” or “poor” water quality for the entire period of study (Pavlidou *et al.,* 2014; Simboura *et al.,* 2014). Therefore, station S2 during the period P-01 (earlier data are not available) was chosen as representative of the “non-good status” (S2 P-01), whereas station S11 during period P-02 was chosen as representative of “good status” (S11 P-02). Of the total 139 samples, the “non-good” conditions included 20 samples and the “good” conditions 8 samples. The remaining 111 were left to be evaluated later.

### Ecological indices

Twenty-seven ecological indices, already used in community ecology and water quality assessment in previous studies (Washington, 1984; Karydis and Tsirtsis, 1996; Spatharis and Tsirtsis, 2010), were screened for their sensitivity to discriminate the “good” and “non-good” conditions (S2 P-0 and S11 P-02). Among those indices, 17 are relevant to diversity, 7 to evenness and 2 to dominance of the mesozooplankton assemblages. Total abundance (whole number of individuals per cubic meter) of each sample was also used as a demo index (Supplementary Table A.I).

### Statistical analyses

Possible differences of each index between the “good” and “non-good” conditions (S2 P-0 and S11 P-02) were tested by applying t-test (significance level alpha = 0.05, *P*-value < 0.05). Its assumptions of normality and homoscedasticity were checked prior to the analyses (Shapiro’s and Bartlett’s tests; for both cases significance level alpha = 0.05, *P*-value > 0.05) and in case they were not met, the non-parametric Wilcoxon test was applied (significance level alpha = 0.05, *P*-value < 0.05).

PCA was then used to combine sensitive indices and identify possible common trends of alpha diversity changes along the two quality levels (e.g. Clarke, 1993; Lundberg *et al.,* 2005; Primpas *et al.,* 2010). Prior to the analysis, the various indices were standardized to equally contribute to the principal components.

Finally, LDA was applied for selected sensitive indices, fulfilling the necessary assumptions, to develop a scale distinguishing the two water quality levels. This scale can be used for the characterization of the water quality of unknown samples, either existing or taken in the future.

Aiming to assess the accuracy of LDA classification, two methods were applied: repeated *k*-fold cross validation and out-of-bootstrap estimate. In *k*-fold cross validation (resampling without replacement) training and test datasets were produced in a systematic way so that after a pre-specified number *k* of subsets (in our case *k* = 5), each of the *n* original cases was left out exactly once. For each subset held out, the model was trained on all other subsets. Accuracy was determined for each test dataset. This procedure was repeated 100 times, and an overall accuracy estimate was provided. In bootstrap, on the other hand, resampling was conducted with replacement. Due to this drawing with replacement procedure, a bootstrapped dataset may contain multiple instances of the same original cases and may completely omit other original cases. For this method, the test cases were those omitted from the bootstrap resampled training set. In our study, 1000 resampling iterations were performed, using the out-of-bootstrap estimate method.

All the data manipulation, the statistical analyses and the results’ visualization were performed through the “R” computer language and environment (R Core Team, 2020), using the packages “stats v3.6.2″ (R Core Team, 2020), “devtools” (Wickham and Seidel, 2020), “tidyverse” (Wickham *et al.,* 2019), “ggbiplot,” “ggplot2″ (Wickham *et al.,* 2016), “MASS” (Venables and Ripley, 2002), “caret” (Kuhn, 2020), “car” (Fox and Weisberg, 2019), “klaR” (Weihs *et al.,* 2005), “scales” (Wickham and Seidel, 2020) and “gridExtra” (Auguie, 2017).

## RESULTS

### Description of mesozooplankton community

**Fig. 2:**
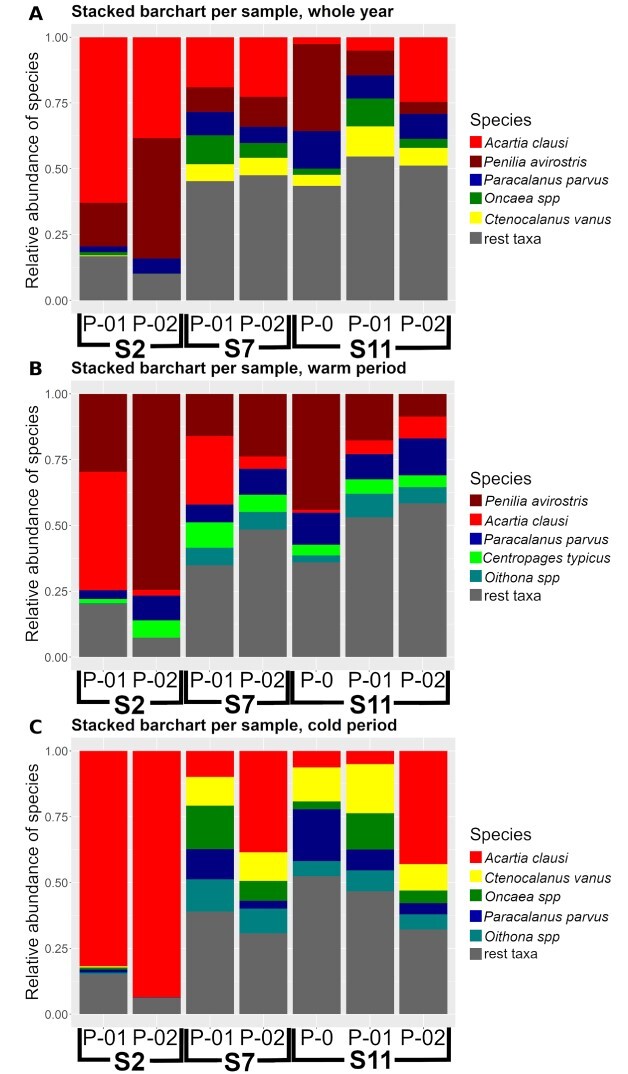
Stacked bar plots of species relative abundance per station and sampling period for (**A**) the whole year, (**B**) warm period and (**C**) cold period. Five most dominant species per period are displayed separately, while the rest are summed together as “rest taxa.” Most abundant “rest taxa” include several species of the genera *Clausocalanus*, *Oithona*, *Temora*, *Evadne*, *Calocalanus*, *Mesocalanus*, *Faranula*, *Corycaeus*, *Podon, Centropages*, *Nannocalanus* and *Paracalanus*.

A total of 139 mesozooplankton samples were used, in which 86 taxa of copepods and cladocerans were identified at species level and occasionally at group of species level. The five most dominant species—per season—of the area are displayed in Fig. 2. Their relative abundances—mainly due to the contribution of only one or two species—appeared much higher at station S2. The abundance of cladocerans was clearly lower during winter, compared both to summer and the whole year. The contribution of cladocerans reached or exceeded 50% of the total abundance at S2 P-02, both during the whole year and the warm period, as well as at S11 station during the warm P-0 period. The most abundant mesozooplankton species—reaching or exceeding 50% of the total abundance per period—consisted of the Calanoida copepods *Acartia (Acartiura) clausi*  Giesbrecht, 1889, *Paracalanus parvus* (Claus, 1863), *Ctenocalanus vanus*  Giesbrecht, 1988, and *Centropages typicus*  Krøyer, 1849; the Cyclopoida copepods *Oncaea* spp*.*  Philippi, 1843, and *Oithona* spp*.*  Baird, 1843*,* and the cladoceran *Penilia avirostris*  Dana, 1849.

The most abundant cladoceran was *P. avirostris,* whereas the most abundant copepod was *A. clausi*. Those two species were always predominant in the S2 station, showing cumulative percentages reaching or exceeding 80% of the total abundance, with *P. avirostris* being significantly more abundant during the warm and *A. clausi* during the cold period. The dominance of those species decreased at stations S7 and S11, moving away from the Psittalia sewage outfall, leading to more even assemblages. An exception was observed for—the early years, of untreated sewage—S11 P-0 throughout the year and during the warm period, where *P. avirostris* showed a contribution of 32–42% to the total abundance. Moreover, during the cold P-02 period of S2 station *A. clausi* contributed approximately to 40% of the total abundance.

Regarding the remaining occasionally dominant species, *P. parvus* was the one (other than *A. clausi* and *P. avirostris*) that remained abundant in all three different seasons, ranging from 8.50 to 21% of the total abundance (depending on the sample and season). There was a clear pattern of reduction in *P. parvus* numbers, in all stations from past (P-0) to recent (P-02) years, during the cold period (Fig. 2C). *Centropages typicus* appeared to be between the most abundant ones only during the warm period, with relative abundances ranging from 1.50 to 9.30%. On the other hand, *Oncaea* spp. were detected as relatively abundant during the whole year (relative abundance 1.50–12.50%) and the cold period (relative abundance 1.50–15%), whereas *Oithona* spp. appeared to be quite abundant during the warm period (1.50–9.30%) and the cold period (1–12.50%). Both of these last taxa had little to no representatives at the heavily burdened S2 station, regardless of the season, or the period.

### Screening of ecological indices

Screening results of 27 ecological indices for their sensitivity to discriminate changes in mesozooplankton alpha diversity, due to water quality, are shown in Supplementary Tables A.II and A.III. Out of 27, 25 indices showed statistically significant differences between “good” and “non-good” conditions, either with parametric tests (where the assumptions of normality and homoscedasticity were met) or with non-parametric methods. Indeed, those differences were statistically significant for 22 out of these 25 indices, both during the whole year and the two periods of the year (warm and cold).

**Fig. 3:**
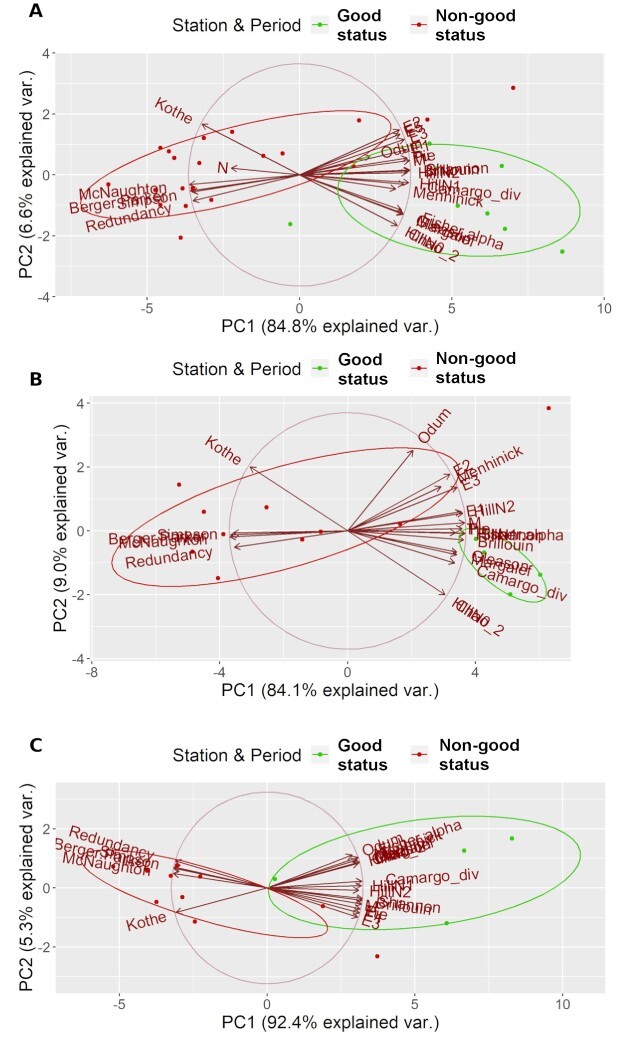
PCA plots produced based on biodiversity indices, baring statistically different values between the reference samples, based on non-parametric Wilcoxon test for (**A**) the whole year, (**B**) warm period and (**C**) cold period.

In general (Fig. 3), out of the diversity indices, identifying with statistical significance (95% confidence) the tested samples, 19 indices (“Margalef,” “Gleason,” “Menhinic,” “Shannon,” “HillN0,” “HillN1,” “HillN2,” “Chao2,” “Odum,” “Pie,” “M,” “Tu,” “Brillouin,” “Camargo Diversity,” “Fisher’s alpha,” “E1,” “E2,” “E3,” “E5”) increased toward the “good” quality direction (S11 P-02 sample). On the other hand, five indices (“Simpson,” “Kothe,” “Redundancy,” “Berger-Parker,” “McNaughton”) showed high values in the direction of the “non-good” quality (S2 P-01 sample).

### Principal component analysis

Sensitive indices able to discriminate the tested conditions (Supplementary Table A.III) were used in a PCA, and the results are shown in Fig. 3. For all the time periods tested (whole year, warm and cold period), the two first principal components explained more than 90% of the variation. Specifically, 91.4% variation was explained using data for the whole year (Fig. 3A), 93.1% for the warm period (Fig. 3B) and 97.7% for the cold period (Fig. 3C).

Overall, most indices expressing diversity and evenness showed an increase toward the “good” status (S11 P-02). Exception only occured for the diversity indices “Kothe” and “Simpson” and the evenness index “Redundancy,” which showed the opposite trend. The same holds for the dominance indices, McNaughton and Berger-Parker, which increased toward the direction of the “non-good” status (S2 P-01).

### Linear discriminant analysis

Only three of the previously displayed indices, namely “HillN0,” “Chao 2” and “Kothe,” were found both sensitive to discriminate the two tested conditions and they also fulfilled the prerequisites (normality and homoscedasticity) for the application of LDA, toward the development of a quality index based on mesozooplankton diversity. Results for the LDA are shown below.

The LDA’s function produced for the whole year is described by the following equation:

$$\kern-6pt\begin{array}{lc} \mathrm{LDA}\ \mathrm{value}\kern-6pt&={0.0907477}^{\ast }\ \mathrm{HillN}0+{0.0353916}^{\ast }\ \mathrm{Kothe}\\&+{0.0907477}^{\ast }\ \mathrm{Chao}2 \end{array}$$

**Fig. 4:**
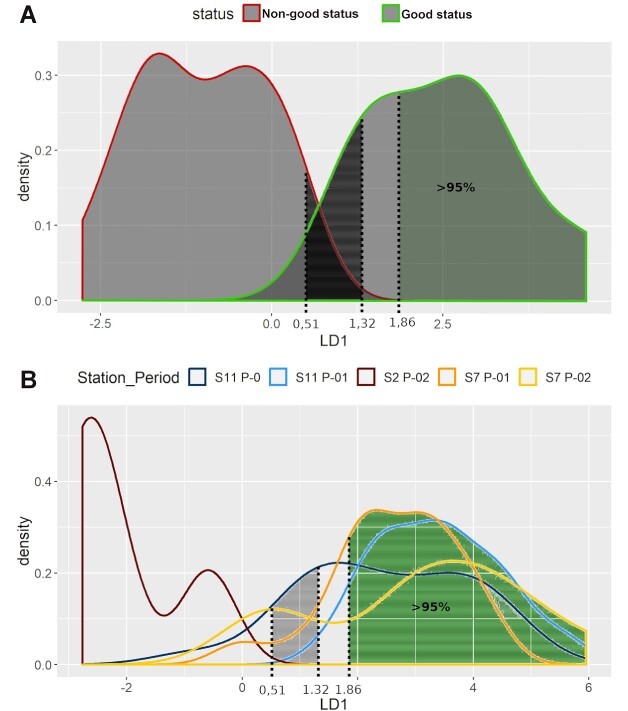
Annual data*.* (**A**) LDA density curves for the two tested conditions: S2 P-01 (non-good status) and S11 P-02 (good status). (**B**) Densities per station and period, of the rest 111 samples, according to the LDA function.

Density curves of LDA values for the two tested conditions on an annual basis are shown in Fig. 4A. The accuracy of our model was 1.00 when estimated with the repeated *k*-fold cross validation method and 0.99 with the out-of-bootstrap estimate method. However, we did observe a small overlap of the two density curves. The lowest LDA value of the “good” status reference condition was 1.32, whereas the corresponding value for the “non-good” condition was 0.51. Samples with LDA values >1.86 were characterized as of “good” status with a probability (posterior probability) of a correct classification >95%. The prior probabilities used for the analysis were set equal to 0.71 for the “non-good” status and 0.29 for the “good” status, resulting in an even stricter “rule” in the classification of samples as of “good” status (Ripley *et al.,* 2018).

The LDA function was then used to evaluate the rest 111 samples (Fig. 4B and Supplementary Table A.IV). Ninety-nine samples of them (90%) were classified with a probability (posterior probability) of correct classification >95%. Specifically, six of them, four samples from S2 P-02 (100%), one from S7 P-01 (5.30%) and one from S11 P-0 (4.50%), were classified in the “non-good” status. Of the rest 93 samples, 17 from S7 P-01 (89%), 9 from S7 P-02 (75%), 15 from S11 P-0 (68%) and 52 from S11 P-01 (96%) were classified in the “good” status.

**Fig. 5:**
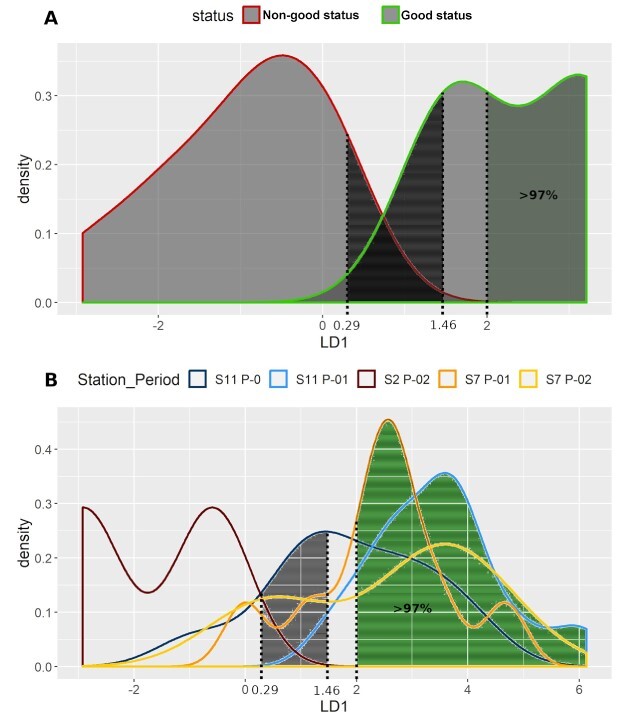
Warm period data. (**A**) LDA density curves for the two reference conditions: S2 P-01 (non-good status) and S11 P-02 (good status). (**B**) Densities per station and period, of the rest 50 samples, according to the LDA function.

**Fig. 6:**
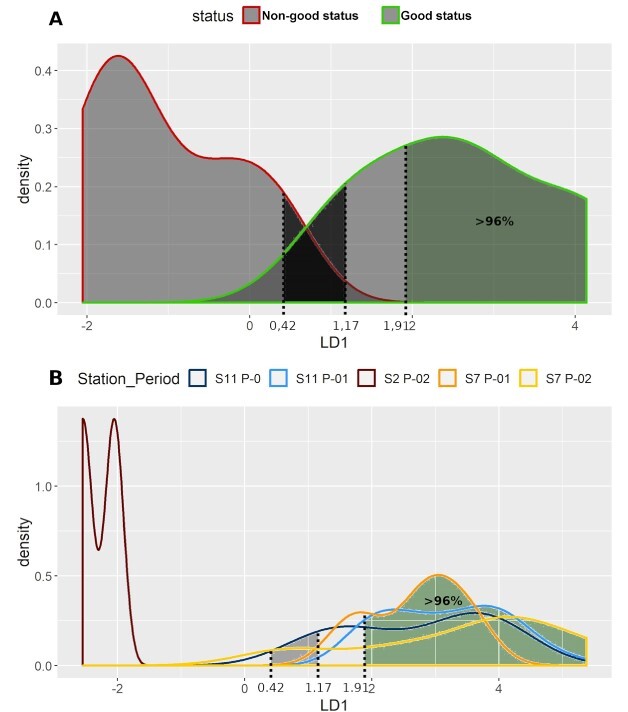
Cold period data. (**A**) LDA density curves for the two reference conditions, S2 P-01 (non-good status) and S11 P-02 (good status). (**B**) Densities per station and period, of the rest 61 samples, according to the LDA function.

LDA was also performed separately per season (warm and cold). The results are shown in Figs 5 and 6, respectively. In the case of warm season (Fig. 5A), the resulting coefficients for the LDA function were 0.097 for HillN0, −0.038 for Kothe and 0.097 for Chao 2. The lowest LDA value of the “good” status samples was 1.46, whereas that of the “non-good” status was 0.29. Samples with LDA values >2 were characterized as of “good” status with posterior probability of correct classification >97%. In the case of warm period, the accuracy of our model was also 1.00 when estimated with the repeated *k*-fold cross validation method and 0.99 with the out-of-bootstrap estimate method.

For the cold period, the coefficients were 0.082 for HillN0, −0.032 for Kothe and 0.082 for Chao 2. The lowest LDA value of the “good” quality conditions was 1.17 and that of the “non-good” quality conditions was 0.42. Samples with LDA values >1.91 were characterized as of “good” condition with a posterior probability of correct classification higher than 96%. In this case, the accuracy of our model was 0.96 when estimated with the repeated *k*-fold cross validation method and 0.95 with the out-of-bootstrap estimate method—a bit lower than the other cases, but still remarkably high.

The two LDA functions produced were then used to evaluate the rest 111 samples (50 from the warm period and 61 from the cold period). The results are presented in Figs 5B and 6B and Supplementary Tables A.V and A.VI, respectively. The combined results of the two seasonal LDAs placed 94%—or else 85%—of the remaining samples in the two quality groups with a posterior probability of correct classification higher than 95%. The results were the same as the annual data, as far as the warm period was concerned. Similar trend was observed for the cold period, except for five samples—two from S7 P-01, two from S11 P-0 and one from S11 P-01—that failed to achieve the 95% threshold. Nonetheless, they were all characterized as of “good” status—as for the annual data - but with a 90% probability of correct classification.

## DISCUSSION

Our hypothesis was that zooplankton alpha diversity indices can express mesozooplankton diversity changes along different water qualities. Based on our results (Screening of ecological indices and Principal component analysis sections), this main hypothesis was confirmed. Specifically, in the case of the ecologically burdened reference sample (S2 P-01), zooplankton species richness decreased, while species evenness decreased and/or dominance of tolerant species (mainly copepod *A. clausi* and cladoceran *P. avirostris*) increased. On the contrary, when we examined the less impacted reference sample (S11 P-02), species richness and evenness metrics were significantly increased. Previous studies (Beaugrand, 2005; Beaugrand *et al.,* 2010; HELCOM, 2012; Aare *et al.,* 2014; Serranito *et al.,* 2016; Villarino *et al.,* 2020) have used ecological indices, expressing diversity, evenness or dominance of zooplankton communities, for seawater quality assessment. However, none of them examined, at the same time, such a variety of widely used biodiversity indices. Other studies have focused on the identification of species, being more sensitive to express human pressures (e.g. Arfi *et al.,* 1981; Siokou-Frangou and Papathanassiou, 1991; Uriarte and Villate, 2005; Kršinić *et al.,* 2007). In our case, we also found that the most abundant species at the most impacted station (S2) and/or during the period of untreated sewage (P0) included the—often characterized as pollution tolerant (Drira *et al.,* 2017), or even opportunistic (Siokou-Frangou and Papathanassiou, 1991)—*A. clausi*, *P. avirostris*, *Oithona* spp. and *P. parvus*. Nevertheless, we chose not to result in the characterization of a particular species or taxa, as indicative of areas under human pressures, but rather create a more holistic qualification method (Linear discriminant analysis section) based on the combination of three zooplankton ecological indices. Such a combination of biodiversity indices is not usual in zooplankton-based studies (Varkitzi *et al.,* 2018). However, it is not uncommon in phytoplankton studies. A similar to ours—but phytoplankton based—recent study recommended the combined use of three indices for the assessment of pelagic habitats: the Shannon’s or Simpson’s Diversity in combination with Sheldon’s Evenness and one of the dominance indices (Francé *et al.,* 2021).

In most zooplankton-based studies, only the total/relative abundance or biomass of total zooplankton/specific taxa has been used to develop water-quality indices (Varkitzi *et al.,* 2018). Especially in the Mediterranean Sea, very scarce studies have provided some quantitative baselines and thresholds for plankton communities, mainly based on the annual mean of mesozooplankton biomass (Varkitzi *et al.,* 2018). What is more, none of the available zooplankton-based state indicators for assessing GES are designed or have defined thresholds for the Mediterranean Sea (Magliozzi *et al.,* 2021). Consequently, we regard the present study as a useful addition to the ongoing research of this domain. One of those few which attempted to identify potential indicators of anthropogenic pressure, using generic indicators of diversity along with ratios of copepod abundances, was the work of Serranito *et al.* (2016) in the Toulon region of Western Mediterranean. Our results on “E1” (Piélou’s evenness) are meeting the above study, as a satisfactory distinctive index between two regions of different quality waters. In several cases of our study, other Evenness’ indices gave statistically significant results as well (“E1,” “E2,” “E3” and “E5”) (Screening of ecological indices section). However, species richness (“HillN0”), instead of an evenness index, was one of the three “key” ecological indices that was selected to construct the linear discriminant analysis (Figs 4–6). This index, or differentiated versions of it, has also been found appropriate at zooplankton-based ecological studies of the North Sea, the extra tropical North Atlantic Ocean (Beaugrand, 2005; Beaugrand *et al.,* 2010) and the Baltic Sea (HELCOM, 2012).

Diversity is multidimensional, especially when viewed as a quantification of difference. Franklin (1988), Noss (1990) and Lyashevska and Farnsworth (2012), suggested that biodiversity is essentially 3D, consisting of structural, taxonomic (a substitute for genetic) and functional diversity. Lyashevska and Farnsworth (2012), also supported that species richness missed a large percentage of the examined biodiversity. In our case, we only dealt with structural diversity—which in relevance to their categorization, is based on different combinations of species numbers and abundances—and we faced no problems in achieving our objectives. Moreover, one of the “key” indices that seemed to work, both to discriminate reference samples and to evaluate others, was, in fact, the number of species “HillN0” (Screening of ecological indices, Principal component analysis and Linear discriminant analysis sections). This could be explained by the fact that we only examined specific key mesozooplankton-groups of the area’s marine ecosystem (copepods and cladocerans), with all its representatives sharing the same ecological role, while the aforementioned studies referred to whole ecological communities.

The proposed linear discriminant analysis (LDA) method in this study was constructed by the combination of three indices: “HillN0,” “Kothe” and “Chao 2.” Apart from the number of species (“HillN0”), both “Chao 2” and “Kothe” have been reported to be either a robust estimator of minimum richness (Shen *et al.,* 2003) or particularly useful in indicating the consequences of point sources of wastewater respectively—especially when combined with other methods of evaluation (Schwoerbel, 1970; Kumar, 2006). LDA was capable of a 100% accurate classification of the two reference samples (Linear discriminant analysis section). The same happened with the classification of the remaining samples of the area; 99 out of a total 111 samplings were classified—with a 95% probability of correct placement—as coming from an area of “good” or “non-good” environmental status (Linear discriminant analysis section).

The LDA produced for the whole year proved to be effective for the characterization of the study area samplings, without the detection of any severe bias due to the seasonal difference among the samplings (Figs 5 and 6). An existence of severe quality differentiation was more than obvious—both from our reference samples and the rest of the area samplings—both in terms of space (from the most heavily burdened S2 to the outer S11, through the intermediate S7) and time (periods P-0 → P-01 → P-02). Furthermore, it is a method based on incidence (presence—absence) data, which is considered as an easier assessment tool. It was not possible to include in our analyses an evenness and/or a dominance index as well, because the obligatory conditions of normal distribution and homoskedasticity of data were not met in several cases of those indices (Screening of ecological indices section). Nonetheless, dominance indices were significantly increased in the case of the “non-good” reference sample (S2 P-01). Moreover, evenness indices showed, in general, higher values in the case of “good status” reference sample (S11 P-02). The trend of these two groups of indices (evenness and dominance) helped in verifying the results of our proposed LDA-based index, despite our inability to include them in an LDA (Principal component analysis section, Fig. 3).

The confirmation of our initial hypothesis, as well as the accuracy of the LDA method, was based on the use of species level data (Principal component analysis section) compared to other studies that have used higher taxonomic levels data to provide biodiversity information. Serranito *et al.* (2016) used the family level as a base for biodiversity estimation. Nevertheless, studies that focused on comparing results of species-level data to those of higher taxa have proposed that only genus-level data are sometimes adequate, but not as robust as the species-level ones (e.g. Vieira *et al.,* 2017; Xiong *et al.,* 2018). Moreover, Gaston (2000) argued that biodiversity information at species level is far more comprehensive and reliable, especially for ecological studies. Consequently, we claim that a species-level-based approach, although more time consuming and demanding a higher level of expertise, ensured the most in-depth and accurate quality assessment of the study area’s waters.

The dominant species in “non-good” status reference sample (S2 P-01) were the cladoceran *P. avirostris*—especially during summer—and the copepod *A. clausi* (Description of mesozooplankton community section) during the cold period. Numerous zooplankton studies in Mediterranean coastal waters confirm the dominance of these two species, with significant tolerance to anthropogenic pressures and strong tendency to dominate the water column (e.g. Arfi *et al.,* 1981; Siokou-Frangou and Papathanassiou, 1991; Uriarte and Villate, 2005; Kršinić *et al.,* 2007). However, in Serranito *et al.* (2016), Oithonidae and not Acartiidae was the main group that distinguished the two bays under study, probably due to their use of the different mesh size net (90 μm) for the zooplankton sampling compared to ours (200 μm). In our study *A. clausi* and *P. avirostris* were followed—in terms of abundance—by small copepods belonging to the orders of Calanoida (*P. parvus*, *C. typicus*, *C. vanus*) or Cyclopoida (*Oithona* spp. and *Oncaea* spp.), which are known to generally dominate the epipelagic layers of the Mediterranean Sea (Siokou-Frangou *et al.,* 2010). The abundance of these species, especially *Oithona* spp and *P. parvus*, is often high in disturbed areas (Blanc *et al.,* 1975; Benon *et al.,* 1977; Moraïtou-Apostolopoulou and Ignatiades, 1980; Arfi *et al.,* 1981; Siokou-Frangou and Papathanassiou, 1991). *Centropages typicus* has also been reported to be associated with regions under severe anthropogenic impact (Drira *et al.,* 2017).

However, the answer is never that straightforward, when trying to associate specific zooplankton taxa to low-quality marine waters. For example, Siokou-Frangou and Papathanassiou (1991) characterized *P. parvus* and *C. typicus* as “less tolerant species,” and the—in cases abundant, in our study—Oncaea species have been described as carnivorous whose absence “reflects the perturbation of the zooplankton communities” (Siokou-Frangou and Papathanassiou, 1991). Clearly, the fact that *A. clausi* and *P. avirostris* have been reported to be dominant at low-quality marine waters further support our results, from a more traditional perspective of analyzing mesozooplankton data. However, we suggest that a water quality index based on diversity indices (combining information about species richness, evenness and/or dominance), rather than specific tolerant/non-tolerant species, can be proven more efficient and robust.

## CONCLUSIONS

The present study provides a proof that mesozooplankton diversity indices can discriminate different levels of anthropogenic impacts and in this sense, it can be used as a reliable indicator for marine water quality assessment in pelagic habitats. Good environmental status was characterized by higher numbers of species, high evenness of species, high diversity and low dominance of one or two species, thus representing a balanced community without properties indicative of eutrophication or a degraded environment.

Our approach for water quality assessment based on zooplankton ecological indices provides a methodology of testing and combining several indices widely used in other fields. We suggest that our study can significantly help the development of indices for biodiversity assessment of the zooplankton “key element,” in the context of Descriptor 1 of the MSFD. We argue that being manageable both in our study area and the rest of the Mediterranean can be done either through the direct use of the combination of indices proposed here or by another combination that can be achieved by following the same steps.

## Supplementary Material

Supplementary_material_JPR_fbac067
