# Supplementary material for: Assessing good environmental status through mesozooplankton biodiversity: a step forward
Source: J Plankton Res. 2022 Nov 28;45(1):52–64. doi: 10.1093/plankt/fbac067 (PMC11233992; doi:10.1093/plankt/fbac067)
Supplement: Supplementary_material_JPR_fbac067 [file supplementary_material_jpr_fbac067.docx]

**Supplementary material**

**Table A.1**

Mathematical formulas of the indices used. Variables used in the formulas: *S* = species number in a sampling, *S_max_* = maximum species number in the whole area, *n* = total number of a sampling’s individuals (used, also, by itself as an index), *n_i_* = species’ “*i*” number of individuals in a sampling, *p_i_* = *n*_i_ / *n, n_1_* and *n_2_* = the number of individuals of the first and second most abundant species in a sampling respectively, q_1_ and q_2_ = the number of species observed only once or twice respectively.

| **Index** | **Name in text and formula** | **Reference** |
| --- | --- | --- |
| *A. Diversity indices* | | |
| Simpson’s D | $Simpson=\frac{\sum_{i=1}^{S} n_{i}\times\left( n_{i}-1 \right)}{n\times\left( n-1 \right)}$ | (Ludwig and Reynolds, 1988) |
| Kothe’s species deficit | $Kothe=\frac{S_{max}-S_{i}}{S_{max}}\times100$ | (Pielou, 1975) |
| Odum’s species per 1000 ind. | $Odum=\frac{S\times1000}{n}$ | (Odum *et al.*, 1960) |
| Gleason’s index | $Gleason=\frac{S}{lnn}$ | (Ludwig and Reynolds, 1988) |
| Margalef’s index | $Margalef=\frac{S-1}{lnn}$ | (Margalef, 1958))⁠ |
| Menhinick’s index | $Menhinick=\frac{S}{\sqrt{n}}$ | (Menhinick, 1964) |
| Shannon’s *H΄* | $Shannon=-\sum_{i=1}^{S} \left( n_{i}n\times lnn_{i}n \right)$ | (Ludwig and Reynolds, 1988) |
| Brillouin’s *H΄* | $Brillouin=\frac{1}{n}\times ln\left( \frac{n!}{\prod_{i=1}^{S} n_{i}!} \right)$ | (Ludwig and Reynolds, 1988) |
| Hill’s N0 | $HillN0=S$ | (Ludwig and Reynolds, 1988) |
| Hill’s N1 | $HillN1=exp\left( Shannon'sH \right)$ | (Ludwig and Reynolds, 1988) |
| Hill’s N2 | $HillN2=\frac{1}{\left( Simpson'sD \right)}$ | (Ludwig and Reynolds, 1988) |
| Hulbert’s PIE | $PIE=\frac{n}{\left( n-1 \right)}\times\left( 1-\sum_{i=1}^{S} p_{i}^{2} \right)$ | (Hurlbert, 1971) |
| McIntosh’s index | $M=\frac{\left( n-\sqrt{\sum_{i=1}^{S} n_{i}^{2}} \right)}{\left( n-\sqrt{n} \right)}$ | (McIntosh, 1967) |
| Keefe’s index | $Tu=1-\frac{n}{\left( n-1 \right)}\times\left( \sum_{i=1}^{S} p_{i}^{2}-\frac{1}{n} \right)$ | (Keefe and Bergersen, 1977) |
| Chao’s S_Chao2_ | $Chao2=S+\frac{q_{1}^{2}}{2\times q_{2}}$ | (Chao, 2006) |
| Camargo’s diversity | $CamargoDiversity=S\times\left( 1-\frac{\sum\left\vert p_{i}-p_{j} \right\vert}{S} \right)$ | (Camargo, 1992) |
| Fisher's alpha | $Fisher'salpha=\frac{n\times\left( 1-c \right)}{c}$, “*c* “ estimated from the iterative solution of: $\frac{S}{n}=\frac{\left( 1-c \right)}{c\times\left( -ln\left( 1-c \right) \right)}$ | (Fisher *et al.*, 1943) |
| *B. Evenness indices* | | |
| Pielou’ s evenness | $E1=\frac{Shannon'sH'}{lnS}$ | (Pielou, 1975) |
| Sheldon’s evenness | $E2=\frac{exp\left( Shannon'sH' \right)}{S}$ | (Sheldon, 1969) |
| Ludwig and Reynolds’ evenness 1 | $E3=\frac{exp\left( Shannon'sH' \right)-1}{S-1}$ | (Ludwig and Reynolds, 1988) |
| Ludwig and Reynolds’ evenness 2 | $E4=\frac{1Simpson'sD}{exp\left( Shannon'sH' \right)}$ | (Ludwig and Reynolds, 1988) |
| Ludwig and Reynolds’ evenness 3 | $E5=\frac{\left( 1Simpson'sD \right)-1}{exp\left( Shannon'sH' \right)-1}$ | (Ludwig and Reynolds, 1988) |
| Redundancy | $Redundancy=\frac{Shannon'sH'_{max}-Shannon'sH'}{Shannon'sH'_{max}-Shannon'sH'_{min}}$  where $H'_{max}=lnS$and $H'_{min}=1n\times ln\left( \frac{n!}{n-S+1!} \right)$ | (Patten, 1962) |
| Camargo’s evenness | $CamargoEvenness=1-\sum\frac{\left\vert p_{i}-p_{j} \right\vert}{S}$ | (Camargo, 1992) |
| *C. Dominance indices* | | |
| Berger-Parker | $Berger\text{-}Parker=\frac{n_{1}}{n}$ | (Berger and Parker, 1970) |
| McNaughton’s index | $McNaughton=\frac{n_{1}+n_{2}}{n}$ | (McNaughton, 1967) |

**Table A.2**

Biodiversity indices’ values, station, period and date, per sampling.

| Sampling | Season | Station &  period | Margalef | Gleason | Menhinick | Simpson | Shannon | HillN0 | HillN1 | HillN2 | E1 | E2 | E3 | E4 | E5 | Odum | Redundancy | Pie | M | Tu | Kothe | Berger-Parker | Brillouin | n | McNaughton | Camargo Evenness | Camargo Diversity | Chao 2 | Fisher’s alpha |
| --- | --- | --- | --- | --- | --- | --- | --- | --- | --- | --- | --- | --- | --- | --- | --- | --- | --- | --- | --- | --- | --- | --- | --- | --- | --- | --- | --- | --- | --- |
| August 1998 | warm | S2 P-01 | 1.694 | 1.815 | 0.241 | 0.193 | 1.931 | 15 | 6.895 | 5.181 | 0.713 | 0.460 | 0.421 | 0.751 | 0.709 | 3.864 | 0.290 | 0.807 | 0.570 | 0.807 | 61.538 | 0.329 | 1.920 | 3882 | 0.527 | 0.943 | 14.142 | 21.125 | 1.978 |
| December 1998 | cold | S2 P-01 | 1.052 | 1.184 | 0.201 | 0.840 | 0.439 | 9 | 1.551 | 1.190 | 0.200 | 0.172 | 0.069 | 0.767 | 0.345 | 4.498 | 0.812 | 0.160 | 0.085 | 0.160 | 76.923 | 0.916 | 0.430 | 2001 | 0.941 | 0.186 | 1.672 | 15.125 | 1.215 |
| February 1999 | cold | S2 P-01 | 2.341 | 2.487 | 0.557 | 0.126 | 2.291 | 17 | 9.882 | 7.930 | 0.809 | 0.581 | 0.555 | 0.803 | 0.780 | 18.280 | 0.200 | 0.874 | 0.665 | 0.874 | 56.410 | 0.214 | 2.249 | 930 | 0.371 | 0.842 | 14.320 | 23.125 | 2.954 |
| May 1999 | warm | S2 P-01 | 1.281 | 1.397 | 0.164 | 0.814 | 0.447 | 12 | 1.564 | 1.229 | 0.180 | 0.130 | 0.051 | 0.786 | 0.407 | 2.233 | 0.826 | 0.186 | 0.099 | 0.186 | 69.231 | 0.899 | 0.442 | 5374 | 0.972 | 0.175 | 2.097 | 18.125 | 1.462 |
| May 2000 | warm | S2 P-01 | 0.844 | 0.964 | 0.126 | 0.719 | 0.671 | 8 | 1.957 | 1.390 | 0.323 | 0.245 | 0.137 | 0.710 | 0.408 | 1.994 | 0.682 | 0.281 | 0.154 | 0.281 | 79.487 | 0.844 | 0.666 | 4012 | 0.923 | 0.262 | 2.094 | 14.125 | 0.959 |
| August 2000 | warm | S2 P-01 | 0.940 | 1.057 | 0.128 | 0.505 | 1.088 | 9 | 2.968 | 1.979 | 0.495 | 0.330 | 0.246 | 0.667 | 0.497 | 1.810 | 0.508 | 0.495 | 0.293 | 0.495 | 76.923 | 0.694 | 1.083 | 4972 | 0.802 | 0.960 | 8.638 | 15.125 | 1.065 |
| December 2000 | cold | S2 P-01 | 1.112 | 1.251 | 0.247 | 0.654 | 0.766 | 9 | 2.151 | 1.529 | 0.349 | 0.239 | 0.144 | 0.711 | 0.460 | 6.767 | 0.664 | 0.346 | 0.197 | 0.346 | 76.923 | 0.799 | 0.752 | 1330 | 0.914 | 0.286 | 2.570 | 15.125 | 1.298 |
| March 2001 | cold | S2 P-01 | 0.738 | 0.861 | 0.120 | 0.737 | 0.595 | 7 | 1.814 | 1.357 | 0.306 | 0.259 | 0.136 | 0.748 | 0.438 | 2.062 | 0.699 | 0.263 | 0.144 | 0.263 | 82.051 | 0.854 | 0.591 | 3394 | 0.928 | 0.268 | 1.875 | 13.125 | 0.843 |
| Μay 2001 | warm | S2 P-01 | 0.686 | 0.857 | 0.270 | 0.814 | 0.439 | 5 | 1.552 | 1.229 | 0.273 | 0.310 | 0.138 | 0.792 | 0.415 | 14.620 | 0.759 | 0.186 | 0.103 | 0.186 | 87.179 | 0.901 | 0.418 | 342 | 0.942 | 0.280 | 1.398 | 11.125 | 0.830 |
| August 2001 | warm | S2 P-01 | 1.496 | 1.621 | 0.236 | 0.392 | 1.430 | 13 | 4.177 | 2.552 | 0.557 | 0.321 | 0.265 | 0.611 | 0.488 | 4.269 | 0.448 | 0.608 | 0.381 | 0.608 | 66.667 | 0.603 | 1.419 | 3045 | 0.711 | 0.943 | 12.259 | 19.125 | 1.741 |
| December 2001 | cold | S2 P-01 | 2.454 | 2.617 | 0.753 | 0.264 | 1.801 | 16 | 6.056 | 3.790 | 0.650 | 0.378 | 0.337 | 0.626 | 0.552 | 35.398 | 0.378 | 0.736 | 0.509 | 0.736 | 58.974 | 0.465 | 1.737 | 452 | 0.622 | 0.545 | 8.719 | 22.125 | 3.234 |
| February 2002 | cold | S2 P-01 | 1.472 | 1.595 | 0.221 | 0.737 | 0.618 | 13 | 1.856 | 1.358 | 0.241 | 0.143 | 0.071 | 0.732 | 0.418 | 3.751 | 0.767 | 0.263 | 0.144 | 0.263 | 66.667 | 0.852 | 0.610 | 3466 | 0.952 | 0.213 | 2.773 | 19.125 | 1.707 |
| June 2002 | warm | S2 P-01 | 1.310 | 1.441 | 0.242 | 0.712 | 0.755 | 11 | 2.127 | 1.405 | 0.315 | 0.193 | 0.113 | 0.660 | 0.359 | 5.317 | 0.696 | 0.288 | 0.160 | 0.288 | 71.795 | 0.841 | 0.743 | 2069 | 0.879 | 0.234 | 2.578 | 17.125 | 1.525 |
| September 2002 | warm | S2 P-01 | 1.391 | 1.507 | 0.174 | 0.416 | 1.275 | 13 | 3.579 | 2.405 | 0.497 | 0.275 | 0.215 | 0.672 | 0.545 | 2.328 | 0.507 | 0.584 | 0.360 | 0.584 | 66.667 | 0.607 | 1.269 | 5585 | 0.803 | 0.958 | 12.452 | 19.125 | 1.593 |
| December 2002 | cold | S2 P-01 | 1.267 | 1.408 | 0.287 | 0.756 | 0.642 | 10 | 1.900 | 1.323 | 0.279 | 0.190 | 0.100 | 0.696 | 0.359 | 8.217 | 0.738 | 0.244 | 0.134 | 0.244 | 74.359 | 0.868 | 0.626 | 1217 | 0.906 | 0.216 | 2.162 | 16.125 | 1.491 |
| February 2003 | cold | S2 P-01 | 0.888 | 0.999 | 0.100 | 0.561 | 0.922 | 9 | 2.514 | 1.784 | 0.420 | 0.279 | 0.189 | 0.710 | 0.518 | 1.101 | 0.583 | 0.439 | 0.254 | 0.439 | 76.923 | 0.731 | 0.919 | 8172 | 0.852 | 0.350 | 3.150 | 15.125 | 0.999 |
| June 2003 | warm | S2 P-01 | 1.780 | 1.898 | 0.237 | 0.808 | 0.551 | 16 | 1.735 | 1.237 | 0.199 | 0.108 | 0.049 | 0.713 | 0.322 | 3.497 | 0.809 | 0.192 | 0.102 | 0.192 | 58.974 | 0.898 | 0.544 | 4575 | 0.922 | 0.154 | 2.467 | 22.125 | 2.079 |
| September 2003 | warm | S2 P-01 | 3.213 | 3.443 | 1.698 | 0.089 | 2.455 | 15 | 11.648 | 11.205 | 0.907 | 0.777 | 0.761 | 0.962 | 0.958 | 192.308 | 0.130 | 0.911 | 0.769 | 0.911 | 61.538 | 0.154 | 2.183 | 78 | 0.308 | 0.856 | 12.846 | 21.125 | 5.522 |
| January 2004 | cold | S2 P-01 | 1.580 | 1.702 | 0.229 | 0.633 | 0.833 | 14 | 2.300 | 1.579 | 0.316 | 0.164 | 0.100 | 0.687 | 0.445 | 3.746 | 0.692 | 0.367 | 0.208 | 0.367 | 64.103 | 0.784 | 0.824 | 3737 | 0.919 | 0.271 | 3.800 | 20.125 | 1.838 |
| March 2004 | cold | S2 P-01 | 0.674 | 0.786 | 0.082 | 0.950 | 0.163 | 7 | 1.177 | 1.053 | 0.084 | 0.168 | 0.030 | 0.894 | 0.298 | 0.953 | 0.920 | 0.050 | 0.026 | 0.050 | 82.051 | 0.975 | 0.161 | 7345 | 0.979 | 0.165 | 1.153 | 13.125 | 0.763 |
| December 2009 | cold | S2 P-02 | 0.906 | 1.057 | 0.255 | 0.268 | 1.464 | 7 | 4.322 | 3.732 | 0.752 | 0.617 | 0.554 | 0.863 | 0.822 | 9.309 | 0.255 | 0.732 | 0.500 | 0.732 | 82.051 | 0.380 | 1.443 | 752 | 0.626 | 0.789 | 5.524 | 13.125 | 1.067 |
| March 2010 | cold | S2 P-02 | 0.416 | 0.520 | 0.041 | 0.942 | 0.162 | 5 | 1.176 | 1.061 | 0.101 | 0.235 | 0.044 | 0.902 | 0.347 | 0.331 | 0.901 | 0.058 | 0.029 | 0.058 | 87.179 | 0.971 | 0.162 | 15085 | 0.988 | 0.224 | 1.118 | 11.125 | 0.483 |
| July 2010 | warm | S2 P-02 | 1.506 | 1.631 | 0.242 | 0.571 | 0.958 | 13 | 2.606 | 1.751 | 0.373 | 0.200 | 0.134 | 0.672 | 0.468 | 4.498 | 0.635 | 0.429 | 0.249 | 0.429 | 66.667 | 0.741 | 0.948 | 2890 | 0.876 | 0.875 | 11.381 | 19.125 | 1.755 |
| August 2010 | warm | S2 P-02 | 0.401 | 0.502 | 0.034 | 0.577 | 0.865 | 5 | 2.374 | 1.733 | 0.537 | 0.475 | 0.344 | 0.730 | 0.533 | 0.235 | 0.463 | 0.423 | 0.242 | 0.423 | 87.179 | 0.746 | 0.864 | 21238 | 0.847 | 0.995 | 4.977 | 11.125 | 0.466 |
| December 1998 | cold | S7 P-01 | 3.501 | 3.635 | 0.659 | 0.127 | 2.481 | 27 | 11.959 | 7.865 | 0.753 | 0.443 | 0.421 | 0.658 | 0.626 | 16.062 | 0.256 | 0.873 | 0.659 | 0.873 | 30.769 | 0.264 | 2.444 | 1681 | 0.436 | 0.820 | 22.140 | 33.125 | 4.568 |
| February 1999 | cold | S7 P-01 | 3.264 | 3.413 | 0.791 | 0.129 | 2.395 | 23 | 10.966 | 7.734 | 0.764 | 0.477 | 0.453 | 0.705 | 0.676 | 27.219 | 0.250 | 0.871 | 0.662 | 0.871 | 41.026 | 0.260 | 2.338 | 845 | 0.425 | 0.773 | 17.787 | 29.125 | 4.363 |
| May 1999 | warm | S7 P-01 | 2.277 | 2.403 | 0.365 | 0.465 | 1.350 | 19 | 3.859 | 2.151 | 0.459 | 0.203 | 0.159 | 0.557 | 0.403 | 7.001 | 0.551 | 0.535 | 0.324 | 0.535 | 51.282 | 0.668 | 1.334 | 2714 | 0.781 | 0.251 | 4.765 | 25.125 | 2.756 |
| May 2000 | warm | S7 P-01 | 2.602 | 2.726 | 0.389 | 0.172 | 2.121 | 22 | 8.336 | 5.826 | 0.686 | 0.379 | 0.349 | 0.699 | 0.658 | 6.886 | 0.319 | 0.828 | 0.596 | 0.828 | 43.590 | 0.298 | 2.103 | 3195 | 0.519 | 0.519 | 11.419 | 28.125 | 3.183 |
| August 2000 | warm | S7 P-01 | 2.985 | 3.115 | 0.510 | 0.393 | 1.555 | 24 | 4.737 | 2.541 | 0.489 | 0.197 | 0.162 | 0.536 | 0.412 | 10.821 | 0.524 | 0.607 | 0.381 | 0.607 | 38.462 | 0.609 | 1.534 | 2218 | 0.727 | 0.948 | 22.747 | 30.125 | 3.761 |
| December 2000 | cold | S7 P-01 | 3.560 | 3.697 | 0.701 | 0.122 | 2.462 | 27 | 11.730 | 8.182 | 0.747 | 0.434 | 0.413 | 0.698 | 0.669 | 18.194 | 0.263 | 0.878 | 0.667 | 0.878 | 30.769 | 0.201 | 2.421 | 1484 | 0.397 | 0.686 | 18.532 | 33.125 | 4.687 |
| March 2001 | cold | S7 P-01 | 3.474 | 3.608 | 0.640 | 0.225 | 1.980 | 27 | 7.241 | 4.439 | 0.601 | 0.268 | 0.240 | 0.613 | 0.551 | 15.186 | 0.413 | 0.775 | 0.538 | 0.775 | 30.769 | 0.416 | 1.948 | 1778 | 0.558 | 0.580 | 15.654 | 33.125 | 4.517 |
| May 2001 | warm | S7 P-01 | 1.717 | 1.839 | 0.254 | 0.639 | 0.947 | 15 | 2.578 | 1.566 | 0.350 | 0.172 | 0.113 | 0.607 | 0.358 | 4.309 | 0.658 | 0.361 | 0.204 | 0.361 | 61.538 | 0.795 | 0.937 | 3481 | 0.849 | 0.247 | 3.702 | 21.125 | 2.012 |
| August 2001 | warm | S7 P-01 | 2.854 | 2.984 | 0.487 | 0.172 | 2.106 | 23 | 8.217 | 5.811 | 0.672 | 0.357 | 0.328 | 0.707 | 0.667 | 10.332 | 0.336 | 0.828 | 0.597 | 0.828 | 41.026 | 0.291 | 2.082 | 2226 | 0.508 | 0.947 | 21.778 | 29.125 | 3.574 |
| December 2001 | cold | S7 P-01 | 4.157 | 4.323 | 1.286 | 0.086 | 2.697 | 26 | 14.833 | 11.580 | 0.828 | 0.570 | 0.553 | 0.781 | 0.765 | 63.570 | 0.194 | 0.914 | 0.739 | 0.914 | 33.333 | 0.161 | 2.582 | 409 | 0.281 | 0.751 | 19.531 | 32.125 | 6.179 |
| February 2002 | cold | S7 P-01 | 4.292 | 4.445 | 1.111 | 0.137 | 2.553 | 29 | 12.851 | 7.294 | 0.758 | 0.443 | 0.423 | 0.568 | 0.531 | 42.584 | 0.263 | 0.863 | 0.653 | 0.863 | 25.641 | 0.326 | 2.472 | 681 | 0.411 | 0.529 | 15.345 | 35.125 | 6.149 |
| June 2002 | warm | S7 P-01 | 4.243 | 4.384 | 0.904 | 0.105 | 2.676 | 31 | 14.530 | 9.539 | 0.779 | 0.469 | 0.451 | 0.656 | 0.631 | 26.338 | 0.233 | 0.895 | 0.695 | 0.895 | 20.513 | 0.243 | 2.619 | 1177 | 0.350 | 0.674 | 20.905 | 37.125 | 5.836 |
| September 2002 | warm | S7 P-01 | 2.971 | 3.095 | 0.441 | 0.174 | 2.241 | 25 | 9.407 | 5.751 | 0.696 | 0.376 | 0.350 | 0.611 | 0.565 | 7.766 | 0.309 | 0.826 | 0.593 | 0.826 | 35.897 | 0.348 | 2.222 | 3219 | 0.521 | 0.788 | 19.708 | 31.125 | 3.692 |
| December 2002 | cold | S7 P-01 | 2.973 | 3.115 | 0.644 | 0.158 | 2.179 | 22 | 8.837 | 6.328 | 0.705 | 0.402 | 0.373 | 0.716 | 0.680 | 18.836 | 0.308 | 0.842 | 0.620 | 0.842 | 43.590 | 0.234 | 2.138 | 1168 | 0.448 | 0.785 | 17.263 | 28.125 | 3.847 |
| February 2003 | cold | S7 P-01 | 3.694 | 3.826 | 0.656 | 0.125 | 2.519 | 29 | 12.417 | 7.979 | 0.748 | 0.428 | 0.408 | 0.643 | 0.611 | 14.819 | 0.260 | 0.875 | 0.660 | 0.875 | 25.641 | 0.230 | 2.483 | 1957 | 0.407 | 0.846 | 24.521 | 35.125 | 4.827 |
| June 2003 | warm | S7 P-01 | 2.878 | 3.003 | 0.441 | 0.155 | 2.255 | 24 | 9.540 | 6.453 | 0.710 | 0.397 | 0.371 | 0.676 | 0.639 | 8.116 | 0.296 | 0.845 | 0.617 | 0.845 | 38.462 | 0.306 | 2.235 | 2957 | 0.440 | 0.668 | 16.021 | 30.125 | 3.571 |
| September 2003 | warm | S7 P-01 | 3.859 | 4.008 | 0.930 | 0.167 | 2.349 | 27 | 10.472 | 5.994 | 0.713 | 0.388 | 0.364 | 0.572 | 0.527 | 32.028 | 0.307 | 0.833 | 0.611 | 0.833 | 30.769 | 0.358 | 2.286 | 843 | 0.485 | 0.884 | 23.880 | 33.125 | 5.324 |
| January 2004 | cold | S7 P-01 | 3.443 | 3.562 | 0.445 | 0.095 | 2.721 | 30 | 15.188 | 10.498 | 0.800 | 0.506 | 0.489 | 0.691 | 0.669 | 6.599 | 0.203 | 0.905 | 0.701 | 0.905 | 23.077 | 0.201 | 2.702 | 4546 | 0.330 | 0.734 | 22.034 | 36.125 | 4.309 |
| March 2004 | cold | S7 P-01 | 2.721 | 2.851 | 0.464 | 0.151 | 2.204 | 22 | 9.066 | 6.622 | 0.713 | 0.412 | 0.384 | 0.730 | 0.697 | 9.800 | 0.294 | 0.849 | 0.624 | 0.849 | 43.590 | 0.230 | 2.181 | 2245 | 0.434 | 0.759 | 16.707 | 28.125 | 3.385 |
| March 2006 | cold | S7 P-02 | 2.010 | 2.128 | 0.262 | 0.573 | 1.117 | 18 | 3.055 | 1.745 | 0.386 | 0.170 | 0.121 | 0.571 | 0.363 | 3.820 | 0.620 | 0.427 | 0.247 | 0.427 | 53.846 | 0.751 | 1.107 | 4712 | 0.808 | 0.282 | 5.073 | 24.125 | 2.370 |
| June 2006 | warm | S7 P-02 | 2.116 | 2.257 | 0.462 | 0.119 | 2.346 | 16 | 10.447 | 8.392 | 0.846 | 0.653 | 0.630 | 0.803 | 0.783 | 13.356 | 0.159 | 0.881 | 0.673 | 0.881 | 58.974 | 0.208 | 2.312 | 1198 | 0.376 | 0.809 | 12.947 | 22.125 | 2.610 |
| September 2006 | warm | S7 P-02 | 2.348 | 2.495 | 0.564 | 0.141 | 2.243 | 17 | 9.422 | 7.088 | 0.792 | 0.554 | 0.526 | 0.752 | 0.723 | 18.681 | 0.218 | 0.859 | 0.644 | 0.859 | 56.410 | 0.254 | 2.201 | 910 | 0.458 | 0.930 | 15.809 | 23.125 | 2.967 |
| December 2006 | cold | S7 P-02 | 3.343 | 3.483 | 0.690 | 0.095 | 2.713 | 25 | 15.075 | 10.550 | 0.843 | 0.603 | 0.586 | 0.700 | 0.679 | 19.069 | 0.164 | 0.905 | 0.711 | 0.905 | 35.897 | 0.225 | 2.666 | 1311 | 0.326 | 0.775 | 19.375 | 31.125 | 4.383 |
| March 2007 | cold | S7 P-02 | 4.582 | 4.729 | 1.086 | 0.091 | 2.846 | 32 | 17.220 | 11.004 | 0.821 | 0.538 | 0.523 | 0.639 | 0.617 | 36.866 | 0.192 | 0.909 | 0.721 | 0.909 | 17.949 | 0.192 | 2.767 | 868 | 0.372 | 0.807 | 25.813 | 38.125 | 6.535 |
| June 2007 | warm | S7 P-02 | 3.384 | 3.519 | 0.647 | 0.120 | 2.445 | 26 | 11.525 | 8.300 | 0.750 | 0.443 | 0.421 | 0.720 | 0.694 | 16.079 | 0.259 | 0.880 | 0.669 | 0.880 | 33.333 | 0.236 | 2.408 | 1617 | 0.399 | 0.755 | 19.625 | 32.125 | 4.400 |
| September 2007 | warm | S7 P-02 | 3.238 | 3.367 | 0.548 | 0.227 | 2.159 | 26 | 8.665 | 4.397 | 0.663 | 0.333 | 0.307 | 0.507 | 0.443 | 11.530 | 0.346 | 0.773 | 0.534 | 0.773 | 33.333 | 0.448 | 2.132 | 2255 | 0.540 | 0.895 | 23.264 | 32.125 | 4.123 |
| December 2007 | cold | S7 P-02 | 4.097 | 4.238 | 0.871 | 0.091 | 2.775 | 30 | 16.031 | 11.048 | 0.816 | 0.534 | 0.518 | 0.689 | 0.669 | 25.295 | 0.194 | 0.909 | 0.719 | 0.909 | 23.077 | 0.191 | 2.717 | 1186 | 0.341 | 0.749 | 22.480 | 36.125 | 5.596 |
| September 2008 | warm | S7 P-02 | 4.140 | 4.293 | 1.074 | 0.082 | 2.705 | 28 | 14.953 | 12.172 | 0.812 | 0.534 | 0.517 | 0.814 | 0.801 | 41.176 | 0.204 | 0.918 | 0.739 | 0.918 | 28.205 | 0.129 | 2.626 | 680 | 0.259 | 0.889 | 24.888 | 34.125 | 5.884 |
| December 2008 | cold | S7 P-02 | 5.509 | 5.662 | 1.410 | 0.065 | 3.044 | 37 | 20.997 | 15.457 | 0.843 | 0.567 | 0.555 | 0.736 | 0.723 | 53.701 | 0.173 | 0.935 | 0.772 | 0.935 | 5.128 | 0.126 | 2.939 | 689 | 0.238 | 0.632 | 23.399 | 43.125 | 8.365 |
| March 2009 | cold | S7 P-02 | 4.853 | 5.004 | 1.221 | 0.115 | 2.604 | 33 | 13.513 | 8.665 | 0.745 | 0.409 | 0.391 | 0.641 | 0.613 | 45.144 | 0.278 | 0.885 | 0.684 | 0.885 | 15.385 | 0.250 | 2.520 | 731 | 0.372 | 0.834 | 27.528 | 39.125 | 7.108 |
| August 2009 | warm | S7 P-02 | 3.883 | 4.017 | 0.717 | 0.238 | 2.059 | 30 | 7.838 | 4.193 | 0.605 | 0.261 | 0.236 | 0.535 | 0.467 | 17.123 | 0.410 | 0.762 | 0.524 | 0.762 | 23.077 | 0.450 | 2.024 | 1752 | 0.576 | 0.866 | 25.987 | 36.125 | 5.142 |
| February 1987 | cold | S11 P-0 | 2.937 | 3.100 | 0.887 | 0.122 | 2.310 | 19 | 10.076 | 8.186 | 0.785 | 0.530 | 0.504 | 0.812 | 0.792 | 41.394 | 0.234 | 0.878 | 0.679 | 0.878 | 51.282 | 0.181 | 2.232 | 459 | 0.353 | 0.761 | 14.468 | 25.125 | 3.998 |
| June 1987 | warm | S11 P-0 | 4.020 | 4.175 | 1.064 | 0.140 | 2.308 | 27 | 10.050 | 7.139 | 0.700 | 0.372 | 0.348 | 0.710 | 0.678 | 41.925 | 0.326 | 0.860 | 0.650 | 0.860 | 30.769 | 0.219 | 2.234 | 644 | 0.435 | 0.560 | 15.127 | 33.125 | 5.701 |
| August 1987 | warm | S11 P-0 | 2.477 | 2.615 | 0.502 | 0.231 | 1.836 | 19 | 6.274 | 4.326 | 0.624 | 0.330 | 0.293 | 0.689 | 0.631 | 13.277 | 0.388 | 0.769 | 0.533 | 0.769 | 51.282 | 0.412 | 1.811 | 1431 | 0.586 | 0.997 | 18.944 | 25.125 | 3.095 |
| September 1987 | warm | S11 P-0 | 2.929 | 3.056 | 0.473 | 0.195 | 1.960 | 24 | 7.099 | 5.139 | 0.617 | 0.296 | 0.265 | 0.724 | 0.679 | 9.320 | 0.392 | 0.805 | 0.570 | 0.805 | 38.462 | 0.267 | 1.940 | 2575 | 0.510 | 0.954 | 22.907 | 30.125 | 3.660 |
| December 1987 | cold | S11 P-0 | 4.705 | 4.861 | 1.278 | 0.105 | 2.557 | 31 | 12.902 | 9.500 | 0.745 | 0.416 | 0.397 | 0.736 | 0.714 | 52.721 | 0.282 | 0.895 | 0.702 | 0.895 | 20.513 | 0.168 | 2.468 | 588 | 0.321 | 0.707 | 21.923 | 37.125 | 6.971 |
| February 1988 | cold | S11 P-0 | 4.462 | 4.616 | 1.163 | 0.098 | 2.600 | 30 | 13.464 | 10.243 | 0.764 | 0.449 | 0.430 | 0.761 | 0.742 | 45.113 | 0.257 | 0.902 | 0.713 | 0.902 | 23.077 | 0.168 | 2.520 | 665 | 0.322 | 0.821 | 24.629 | 36.125 | 6.460 |
| March 1989 | cold | S11 P-0 | 3.628 | 3.785 | 1.008 | 0.402 | 1.458 | 24 | 4.295 | 2.487 | 0.459 | 0.179 | 0.143 | 0.579 | 0.451 | 42.328 | 0.589 | 0.598 | 0.381 | 0.598 | 38.462 | 0.608 | 1.396 | 567 | 0.735 | 0.851 | 20.414 | 30.125 | 5.081 |
| June 1989 | warm | S11 P-0 | 3.599 | 3.749 | 0.891 | 0.155 | 2.263 | 25 | 9.610 | 6.461 | 0.703 | 0.384 | 0.359 | 0.672 | 0.634 | 31.766 | 0.317 | 0.845 | 0.628 | 0.845 | 35.897 | 0.277 | 2.203 | 787 | 0.492 | 0.779 | 19.463 | 31.125 | 4.920 |
| September 1989 | warm | S11 P-0 | 3.136 | 3.286 | 0.773 | 0.157 | 2.086 | 22 | 8.056 | 6.351 | 0.675 | 0.366 | 0.336 | 0.788 | 0.758 | 27.194 | 0.344 | 0.843 | 0.624 | 0.843 | 43.590 | 0.258 | 2.038 | 809 | 0.454 | 0.991 | 21.792 | 28.125 | 4.173 |
| December 1989 | cold | S11 P-0 | 4.725 | 4.873 | 1.117 | 0.137 | 2.417 | 33 | 11.209 | 7.305 | 0.691 | 0.340 | 0.319 | 0.652 | 0.618 | 37.801 | 0.332 | 0.863 | 0.651 | 0.863 | 15.385 | 0.236 | 2.348 | 873 | 0.419 | 0.750 | 24.750 | 39.125 | 6.783 |
| March 1990 | cold | S11 P-0 | 4.229 | 4.375 | 0.973 | 0.132 | 2.379 | 30 | 10.794 | 7.579 | 0.699 | 0.360 | 0.338 | 0.702 | 0.672 | 31.546 | 0.320 | 0.868 | 0.657 | 0.868 | 23.077 | 0.237 | 2.322 | 951 | 0.446 | 0.775 | 23.244 | 36.125 | 5.894 |
| December 1992 | cold | S11 P-0 | 3.355 | 3.515 | 0.962 | 0.138 | 2.345 | 22 | 10.437 | 7.244 | 0.759 | 0.474 | 0.449 | 0.694 | 0.662 | 42.065 | 0.263 | 0.862 | 0.655 | 0.862 | 43.590 | 0.281 | 2.267 | 523 | 0.453 | 0.974 | 21.436 | 28.125 | 4.650 |
| March 1992 | cold | S11 P-0 | 3.636 | 3.770 | 0.683 | 0.129 | 2.341 | 28 | 10.395 | 7.744 | 0.703 | 0.371 | 0.348 | 0.745 | 0.718 | 16.667 | 0.308 | 0.871 | 0.656 | 0.871 | 28.205 | 0.216 | 2.306 | 1680 | 0.399 | 0.737 | 20.648 | 34.125 | 4.773 |
| June 1992 | warm | S11 P-0 | 2.220 | 2.359 | 0.463 | 0.323 | 1.540 | 17 | 4.666 | 3.092 | 0.544 | 0.274 | 0.229 | 0.663 | 0.571 | 12.611 | 0.471 | 0.677 | 0.443 | 0.677 | 56.410 | 0.454 | 1.515 | 1348 | 0.790 | 0.973 | 16.541 | 23.125 | 2.742 |
| September 1992 | warm | S11 P-0 | 2.759 | 2.912 | 0.728 | 0.241 | 1.785 | 19 | 5.958 | 4.154 | 0.606 | 0.314 | 0.275 | 0.697 | 0.636 | 27.859 | 0.418 | 0.759 | 0.528 | 0.759 | 51.282 | 0.409 | 1.737 | 682 | 0.626 | 0.995 | 18.903 | 25.125 | 3.624 |
| December 1992 | cold | S11 P-0 | 3.355 | 3.515 | 0.962 | 0.138 | 2.345 | 22 | 10.437 | 7.244 | 0.759 | 0.474 | 0.449 | 0.694 | 0.662 | 42.065 | 0.263 | 0.862 | 0.655 | 0.862 | 43.590 | 0.281 | 2.267 | 523 | 0.453 | 0.974 | 21.436 | 28.125 | 4.650 |
| March 1993 | cold | S11 P-0 | 2.911 | 3.064 | 0.765 | 0.177 | 2.031 | 20 | 7.625 | 5.641 | 0.678 | 0.381 | 0.349 | 0.740 | 0.701 | 29.283 | 0.343 | 0.823 | 0.601 | 0.823 | 48.718 | 0.269 | 1.978 | 683 | 0.489 | 0.840 | 16.794 | 26.125 | 3.860 |
| September 1993 | warm | S11 P-0 | 1.292 | 1.409 | 0.170 | 0.420 | 1.194 | 12 | 3.299 | 2.378 | 0.480 | 0.275 | 0.209 | 0.721 | 0.599 | 2.404 | 0.524 | 0.580 | 0.357 | 0.580 | 69.231 | 0.604 | 1.188 | 4992 | 0.810 | 0.995 | 11.941 | 18.125 | 1.477 |
| December 1993 | cold | S11 P-0 | 4.379 | 4.547 | 1.387 | 0.138 | 2.391 | 27 | 10.928 | 7.256 | 0.726 | 0.405 | 0.382 | 0.664 | 0.630 | 71.240 | 0.313 | 0.862 | 0.660 | 0.862 | 30.769 | 0.288 | 2.278 | 379 | 0.427 | 0.436 | 11.765 | 33.125 | 6.650 |
| March 1994 | cold | S11 P-0 | 4.599 | 4.752 | 1.188 | 0.084 | 2.739 | 31 | 15.464 | 11.880 | 0.797 | 0.499 | 0.482 | 0.768 | 0.752 | 45.521 | 0.221 | 0.916 | 0.736 | 0.916 | 20.513 | 0.147 | 2.653 | 681 | 0.272 | 0.712 | 22.059 | 37.125 | 6.692 |
| June 1994 | warm | S11 P-0 | 2.146 | 2.259 | 0.239 | 0.469 | 1.292 | 20 | 3.639 | 2.131 | 0.431 | 0.182 | 0.139 | 0.586 | 0.429 | 2.863 | 0.573 | 0.531 | 0.319 | 0.531 | 48.718 | 0.671 | 1.285 | 6986 | 0.747 | 0.995 | 19.907 | 26.125 | 2.523 |
| September 1994 | warm | S11 P-0 | 3.778 | 3.913 | 0.713 | 0.138 | 2.408 | 29 | 11.113 | 7.269 | 0.715 | 0.383 | 0.361 | 0.654 | 0.620 | 17.533 | 0.296 | 0.862 | 0.644 | 0.862 | 25.641 | 0.310 | 2.372 | 1654 | 0.401 | 0.812 | 23.543 | 35.125 | 4.995 |
| February 1995 | cold | S11 P-01 | 3.446 | 3.589 | 0.768 | 0.144 | 2.171 | 25 | 8.768 | 6.958 | 0.674 | 0.351 | 0.324 | 0.794 | 0.767 | 23.607 | 0.342 | 0.856 | 0.639 | 0.856 | 35.897 | 0.212 | 2.127 | 1059 | 0.398 | 0.841 | 21.036 | 31.125 | 4.591 |
| July 1995 | warm | S11 P-01 | 2.634 | 2.759 | 0.408 | 0.280 | 1.909 | 22 | 6.750 | 3.570 | 0.618 | 0.307 | 0.274 | 0.529 | 0.447 | 7.581 | 0.390 | 0.720 | 0.479 | 0.720 | 43.590 | 0.499 | 1.890 | 2902 | 0.620 | 0.962 | 21.153 | 28.125 | 3.235 |
| October 1995 | warm | S11 P-01 | 3.470 | 3.609 | 0.709 | 0.175 | 2.110 | 26 | 8.246 | 5.711 | 0.648 | 0.317 | 0.290 | 0.692 | 0.650 | 19.331 | 0.368 | 0.825 | 0.597 | 0.825 | 33.333 | 0.288 | 2.072 | 1345 | 0.520 | 0.612 | 15.909 | 32.125 | 4.571 |
| December 1995 | cold | S11 P-01 | 4.695 | 4.851 | 1.270 | 0.142 | 2.425 | 31 | 11.298 | 7.029 | 0.706 | 0.364 | 0.343 | 0.622 | 0.585 | 52.013 | 0.324 | 0.858 | 0.647 | 0.858 | 20.513 | 0.297 | 2.337 | 596 | 0.473 | 0.469 | 14.525 | 37.125 | 6.945 |
| March 1997 | cold | S11 P-01 | 4.139 | 4.287 | 0.985 | 0.167 | 2.187 | 29 | 8.907 | 5.990 | 0.649 | 0.307 | 0.282 | 0.673 | 0.631 | 33.449 | 0.375 | 0.833 | 0.611 | 0.833 | 25.641 | 0.302 | 2.130 | 867 | 0.510 | 0.765 | 22.183 | 35.125 | 5.780 |
| December 1997 | cold | S11 P-01 | 3.925 | 4.103 | 1.395 | 0.189 | 2.125 | 23 | 8.374 | 5.301 | 0.678 | 0.364 | 0.335 | 0.633 | 0.583 | 84.559 | 0.376 | 0.811 | 0.599 | 0.811 | 41.026 | 0.298 | 1.998 | 272 | 0.585 | 0.941 | 21.654 | 29.125 | 5.995 |
| February 1998 | cold | S11 P-01 | 3.030 | 3.168 | 0.610 | 0.117 | 2.421 | 23 | 11.252 | 8.561 | 0.772 | 0.489 | 0.466 | 0.761 | 0.738 | 16.174 | 0.236 | 0.883 | 0.675 | 0.883 | 41.026 | 0.211 | 2.383 | 1422 | 0.356 | 0.814 | 18.715 | 29.125 | 3.897 |
| October 1998 | warm | S11 P-01 | 3.675 | 3.806 | 0.642 | 0.126 | 2.454 | 29 | 11.640 | 7.926 | 0.729 | 0.401 | 0.380 | 0.681 | 0.651 | 14.230 | 0.280 | 0.874 | 0.659 | 0.874 | 25.641 | 0.273 | 2.422 | 2038 | 0.401 | 0.859 | 24.899 | 35.125 | 4.789 |
| August 1998 | warm | S11 P-01 | 3.316 | 3.443 | 0.535 | 0.143 | 2.455 | 27 | 11.648 | 6.969 | 0.745 | 0.431 | 0.410 | 0.598 | 0.561 | 10.617 | 0.261 | 0.857 | 0.633 | 0.857 | 30.769 | 0.330 | 2.429 | 2543 | 0.405 | 0.916 | 24.736 | 33.125 | 4.216 |
| December 1998 | cold | S11 P-01 | 3.987 | 4.129 | 0.866 | 0.089 | 2.715 | 29 | 15.103 | 11.259 | 0.806 | 0.521 | 0.504 | 0.745 | 0.727 | 25.847 | 0.204 | 0.911 | 0.722 | 0.911 | 25.641 | 0.169 | 2.659 | 1122 | 0.299 | 0.785 | 22.773 | 35.125 | 5.436 |
| January 1999 | cold | S11 P-01 | 3.297 | 3.434 | 0.656 | 0.090 | 2.728 | 25 | 15.295 | 11.173 | 0.847 | 0.612 | 0.596 | 0.730 | 0.712 | 17.230 | 0.159 | 0.910 | 0.719 | 0.910 | 35.897 | 0.199 | 2.685 | 1451 | 0.307 | 0.875 | 21.864 | 31.125 | 4.291 |
| February 1999 | cold | S11 P-01 | 3.612 | 3.746 | 0.667 | 0.118 | 2.493 | 28 | 12.097 | 8.488 | 0.748 | 0.432 | 0.411 | 0.702 | 0.675 | 15.873 | 0.261 | 0.882 | 0.672 | 0.882 | 28.205 | 0.230 | 2.456 | 1764 | 0.374 | 0.856 | 23.962 | 34.125 | 4.726 |
| March 1999 | cold | S11 P-01 | 4.301 | 4.444 | 0.948 | 0.157 | 2.321 | 31 | 10.181 | 6.366 | 0.676 | 0.328 | 0.306 | 0.625 | 0.584 | 28.972 | 0.344 | 0.843 | 0.622 | 0.843 | 20.513 | 0.293 | 2.264 | 1070 | 0.486 | 0.609 | 18.865 | 37.125 | 5.968 |
| April 1999 | cold | S11 P-01 | 3.030 | 3.156 | 0.476 | 0.158 | 2.308 | 25 | 10.054 | 6.342 | 0.717 | 0.402 | 0.377 | 0.631 | 0.590 | 9.078 | 0.289 | 0.842 | 0.614 | 0.842 | 35.897 | 0.337 | 2.285 | 2754 | 0.442 | 0.584 | 14.593 | 31.125 | 3.794 |
| May 1999 | warm | S11 P-01 | 3.174 | 3.312 | 0.641 | 0.101 | 2.547 | 24 | 12.773 | 9.892 | 0.802 | 0.532 | 0.512 | 0.774 | 0.755 | 17.106 | 0.206 | 0.899 | 0.700 | 0.899 | 38.462 | 0.164 | 2.508 | 1403 | 0.319 | 0.663 | 15.900 | 30.125 | 4.113 |
| June 1999 | warm | S11 P-01 | 3.476 | 3.610 | 0.641 | 0.099 | 2.528 | 27 | 12.533 | 10.051 | 0.767 | 0.464 | 0.444 | 0.802 | 0.785 | 15.237 | 0.241 | 0.901 | 0.700 | 0.901 | 30.769 | 0.167 | 2.494 | 1772 | 0.304 | 0.741 | 20.015 | 33.125 | 4.520 |
| September 1999 | warm | S11 P-01 | 2.537 | 2.670 | 0.473 | 0.304 | 1.811 | 20 | 6.118 | 3.286 | 0.605 | 0.306 | 0.269 | 0.537 | 0.447 | 11.173 | 0.406 | 0.696 | 0.459 | 0.696 | 48.718 | 0.527 | 1.786 | 1790 | 0.621 | 0.975 | 19.505 | 26.125 | 3.153 |
| December 1999 | cold | S11 P-01 | 4.354 | 4.522 | 1.364 | 0.088 | 2.761 | 27 | 15.809 | 11.317 | 0.838 | 0.586 | 0.570 | 0.716 | 0.697 | 68.878 | 0.184 | 0.912 | 0.736 | 0.912 | 30.769 | 0.202 | 2.634 | 392 | 0.342 | 0.760 | 20.531 | 33.125 | 6.579 |
| February 2000 | cold | S11 P-01 | 3.397 | 3.551 | 0.902 | 0.140 | 2.335 | 23 | 10.326 | 7.120 | 0.745 | 0.449 | 0.424 | 0.689 | 0.656 | 35.385 | 0.275 | 0.860 | 0.649 | 0.860 | 41.026 | 0.242 | 2.264 | 650 | 0.429 | 0.731 | 16.812 | 29.125 | 4.649 |
| May 2000 | warm | S11 P-01 | 2.959 | 3.088 | 0.493 | 0.179 | 2.077 | 24 | 7.984 | 5.586 | 0.654 | 0.333 | 0.304 | 0.700 | 0.657 | 10.114 | 0.355 | 0.821 | 0.589 | 0.821 | 38.462 | 0.264 | 2.054 | 2373 | 0.504 | 0.577 | 13.839 | 30.125 | 3.714 |
| June 2000 | warm | S11 P-01 | 3.077 | 3.217 | 0.645 | 0.178 | 2.112 | 23 | 8.263 | 5.612 | 0.673 | 0.359 | 0.330 | 0.679 | 0.635 | 18.068 | 0.340 | 0.822 | 0.594 | 0.822 | 41.026 | 0.322 | 2.074 | 1273 | 0.522 | 0.775 | 17.831 | 29.125 | 3.987 |
| August 2000 | warm | S11 P-01 | 3.796 | 3.932 | 0.726 | 0.214 | 2.116 | 29 | 8.295 | 4.674 | 0.628 | 0.286 | 0.261 | 0.563 | 0.504 | 18.159 | 0.387 | 0.786 | 0.551 | 0.786 | 25.641 | 0.413 | 2.079 | 1597 | 0.553 | 0.957 | 27.757 | 35.125 | 5.032 |
| September 2000 | warm | S11 P-01 | 3.667 | 3.808 | 0.779 | 0.114 | 2.419 | 27 | 11.239 | 8.759 | 0.734 | 0.416 | 0.394 | 0.779 | 0.758 | 22.481 | 0.279 | 0.886 | 0.681 | 0.886 | 30.769 | 0.209 | 2.375 | 1201 | 0.354 | 0.882 | 23.818 | 33.125 | 4.905 |
| December 2000 | cold | S11 P-01 | 4.281 | 4.424 | 0.933 | 0.086 | 2.732 | 31 | 15.368 | 11.660 | 0.796 | 0.496 | 0.479 | 0.759 | 0.742 | 28.054 | 0.216 | 0.914 | 0.728 | 0.914 | 20.513 | 0.146 | 2.673 | 1105 | 0.275 | 0.642 | 19.891 | 37.125 | 5.923 |
| January 2001 | cold | S11 P-01 | 3.866 | 4.009 | 0.852 | 0.105 | 2.607 | 28 | 13.555 | 9.515 | 0.782 | 0.484 | 0.465 | 0.702 | 0.678 | 25.950 | 0.230 | 0.895 | 0.696 | 0.895 | 28.205 | 0.220 | 2.551 | 1079 | 0.348 | 0.870 | 24.364 | 34.125 | 5.254 |
| March 2001 | cold | S11 P-01 | 4.035 | 4.190 | 1.077 | 0.189 | 2.111 | 27 | 8.253 | 5.296 | 0.640 | 0.306 | 0.279 | 0.642 | 0.592 | 42.925 | 0.391 | 0.811 | 0.587 | 0.811 | 30.769 | 0.312 | 2.038 | 629 | 0.571 | 0.914 | 24.676 | 33.125 | 5.737 |
| April 2001 | cold | S11 P-01 | 3.204 | 3.350 | 0.743 | 0.100 | 2.557 | 23 | 12.901 | 9.998 | 0.816 | 0.561 | 0.541 | 0.775 | 0.756 | 23.983 | 0.194 | 0.900 | 0.705 | 0.900 | 41.026 | 0.182 | 2.504 | 959 | 0.327 | 0.884 | 20.341 | 29.125 | 4.239 |
| May 2001 | warm | S11 P-01 | 3.595 | 3.751 | 0.979 | 0.112 | 2.566 | 24 | 13.010 | 8.968 | 0.807 | 0.542 | 0.522 | 0.689 | 0.663 | 39.933 | 0.209 | 0.888 | 0.692 | 0.888 | 38.462 | 0.258 | 2.486 | 601 | 0.364 | 0.782 | 18.762 | 30.125 | 5.003 |
| June 2001 | warm | S11 P-01 | 3.198 | 3.331 | 0.586 | 0.128 | 2.387 | 25 | 10.884 | 7.838 | 0.742 | 0.435 | 0.412 | 0.720 | 0.692 | 13.751 | 0.267 | 0.872 | 0.658 | 0.872 | 35.897 | 0.257 | 2.356 | 1818 | 0.401 | 0.587 | 14.663 | 31.125 | 4.101 |
| August 2001 | warm | S11 P-01 | 2.763 | 2.901 | 0.563 | 0.135 | 2.346 | 21 | 10.445 | 7.381 | 0.771 | 0.497 | 0.472 | 0.707 | 0.676 | 15.075 | 0.238 | 0.865 | 0.648 | 0.865 | 46.154 | 0.235 | 2.310 | 1393 | 0.448 | 0.972 | 20.419 | 27.125 | 3.508 |
| September 2001 | warm | S11 P-01 | 3.700 | 3.837 | 0.729 | 0.126 | 2.417 | 28 | 11.218 | 7.931 | 0.725 | 0.401 | 0.378 | 0.707 | 0.678 | 18.970 | 0.286 | 0.874 | 0.661 | 0.874 | 28.205 | 0.231 | 2.376 | 1476 | 0.415 | 0.887 | 24.847 | 34.125 | 4.903 |
| December 2001 | cold | S11 P-01 | 5.159 | 5.320 | 1.485 | 0.066 | 2.975 | 33 | 19.594 | 15.159 | 0.851 | 0.594 | 0.581 | 0.774 | 0.761 | 66.802 | 0.168 | 0.934 | 0.774 | 0.934 | 15.385 | 0.128 | 2.852 | 494 | 0.243 | 0.734 | 24.223 | 39.125 | 7.964 |
| January 2002 | cold | S11 P-01 | 3.226 | 3.355 | 0.539 | 0.096 | 2.632 | 26 | 13.905 | 10.394 | 0.808 | 0.535 | 0.516 | 0.748 | 0.728 | 11.192 | 0.197 | 0.904 | 0.704 | 0.904 | 33.333 | 0.184 | 2.603 | 2323 | 0.316 | 0.813 | 21.137 | 32.125 | 4.100 |
| March 2002 | cold | S11 P-01 | 4.352 | 4.492 | 0.908 | 0.159 | 2.396 | 32 | 10.980 | 6.295 | 0.691 | 0.343 | 0.322 | 0.573 | 0.531 | 25.786 | 0.325 | 0.841 | 0.618 | 0.841 | 17.949 | 0.341 | 2.344 | 1241 | 0.470 | 0.777 | 24.872 | 38.125 | 5.995 |
| April 2002 | cold | S11 P-01 | 3.494 | 3.646 | 0.893 | 0.095 | 2.638 | 24 | 13.989 | 10.540 | 0.830 | 0.583 | 0.565 | 0.753 | 0.734 | 33.241 | 0.182 | 0.905 | 0.717 | 0.905 | 38.462 | 0.176 | 2.567 | 722 | 0.313 | 0.879 | 21.107 | 30.125 | 4.776 |
| May 2002 | warm | S11 P-01 | 4.834 | 4.995 | 1.392 | 0.127 | 2.527 | 31 | 12.522 | 7.884 | 0.736 | 0.404 | 0.384 | 0.630 | 0.597 | 62.500 | 0.296 | 0.873 | 0.672 | 0.873 | 20.513 | 0.278 | 2.422 | 496 | 0.409 | 0.841 | 26.056 | 37.125 | 7.330 |
| June 2002 | warm | S11 P-01 | 4.558 | 4.692 | 0.840 | 0.129 | 2.488 | 35 | 12.037 | 7.735 | 0.700 | 0.344 | 0.325 | 0.643 | 0.610 | 20.161 | 0.313 | 0.871 | 0.655 | 0.871 | 10.256 | 0.247 | 2.446 | 1736 | 0.433 | 0.724 | 25.346 | 41.125 | 6.209 |
| August 2002 | warm | S11 P-01 | 3.815 | 3.957 | 0.814 | 0.138 | 2.409 | 28 | 11.124 | 7.267 | 0.723 | 0.397 | 0.375 | 0.653 | 0.619 | 23.649 | 0.291 | 0.862 | 0.647 | 0.862 | 28.205 | 0.292 | 2.360 | 1184 | 0.440 | 0.892 | 24.976 | 34.125 | 5.144 |
| September 2002 | warm | S11 P-01 | 3.707 | 3.840 | 0.664 | 0.133 | 2.500 | 29 | 12.177 | 7.530 | 0.742 | 0.420 | 0.399 | 0.618 | 0.584 | 15.215 | 0.266 | 0.867 | 0.650 | 0.867 | 25.641 | 0.303 | 2.464 | 1906 | 0.406 | 0.909 | 26.349 | 35.125 | 4.853 |
| December 2002 | cold | S11 P-01 | 4.054 | 4.189 | 0.766 | 0.129 | 2.451 | 31 | 11.596 | 7.754 | 0.714 | 0.374 | 0.353 | 0.669 | 0.637 | 18.949 | 0.298 | 0.871 | 0.656 | 0.871 | 20.513 | 0.247 | 2.410 | 1636 | 0.429 | 0.773 | 23.957 | 37.125 | 5.427 |
| February 2003 | cold | S11 P-01 | 4.256 | 4.393 | 0.838 | 0.172 | 2.389 | 32 | 10.907 | 5.810 | 0.689 | 0.341 | 0.320 | 0.533 | 0.485 | 21.963 | 0.325 | 0.828 | 0.600 | 0.828 | 17.949 | 0.356 | 2.342 | 1457 | 0.522 | 0.845 | 27.033 | 38.125 | 5.783 |
| March 2003 | cold | S11 P-01 | 4.375 | 4.508 | 0.783 | 0.152 | 2.332 | 34 | 10.298 | 6.570 | 0.661 | 0.303 | 0.282 | 0.638 | 0.599 | 18.028 | 0.352 | 0.848 | 0.624 | 0.848 | 12.821 | 0.296 | 2.294 | 1886 | 0.457 | 0.899 | 30.583 | 40.125 | 5.891 |
| April 2003 | cold | S11 P-01 | 3.349 | 3.465 | 0.395 | 0.140 | 2.343 | 30 | 10.414 | 7.143 | 0.689 | 0.347 | 0.325 | 0.686 | 0.653 | 5.207 | 0.315 | 0.860 | 0.634 | 0.860 | 23.077 | 0.276 | 2.330 | 5761 | 0.446 | 0.896 | 26.873 | 36.125 | 4.145 |
| May 2003 | warm | S11 P-01 | 3.252 | 3.382 | 0.557 | 0.123 | 2.424 | 26 | 11.297 | 8.154 | 0.744 | 0.434 | 0.412 | 0.722 | 0.695 | 11.927 | 0.263 | 0.877 | 0.663 | 0.877 | 33.333 | 0.194 | 2.396 | 2180 | 0.376 | 0.849 | 22.070 | 32.125 | 4.149 |
| June 2003 | warm | S11 P-01 | 3.568 | 3.700 | 0.637 | 0.106 | 2.506 | 28 | 12.256 | 9.469 | 0.752 | 0.438 | 0.417 | 0.773 | 0.752 | 14.485 | 0.256 | 0.894 | 0.690 | 0.894 | 28.205 | 0.178 | 2.474 | 1933 | 0.336 | 0.748 | 20.943 | 34.125 | 4.640 |
| August 2003 | warm | S11 P-01 | 3.817 | 3.958 | 0.815 | 0.136 | 2.416 | 28 | 11.206 | 7.335 | 0.725 | 0.400 | 0.378 | 0.655 | 0.621 | 23.709 | 0.289 | 0.864 | 0.649 | 0.864 | 28.205 | 0.290 | 2.368 | 1181 | 0.438 | 0.892 | 24.975 | 34.125 | 5.147 |
| September 2003 | warm | S11 P-01 | 4.497 | 4.625 | 0.735 | 0.112 | 2.600 | 36 | 13.459 | 8.968 | 0.725 | 0.374 | 0.356 | 0.666 | 0.640 | 15.000 | 0.284 | 0.888 | 0.679 | 0.888 | 7.692 | 0.202 | 2.566 | 2400 | 0.395 | 0.745 | 26.822 | 42.125 | 6.007 |
| October 2003 | warm | S11 P-01 | 4.926 | 5.066 | 1.031 | 0.074 | 2.887 | 36 | 17.935 | 13.475 | 0.806 | 0.498 | 0.484 | 0.751 | 0.737 | 29.532 | 0.206 | 0.926 | 0.748 | 0.926 | 7.692 | 0.138 | 2.825 | 1219 | 0.269 | 0.526 | 18.921 | 42.125 | 6.962 |
| January 2004 | cold | S11 P-01 | 4.645 | 4.790 | 1.053 | 0.087 | 2.834 | 33 | 17.006 | 11.516 | 0.810 | 0.515 | 0.500 | 0.677 | 0.657 | 33.605 | 0.203 | 0.913 | 0.727 | 0.913 | 15.385 | 0.184 | 2.763 | 982 | 0.332 | 0.701 | 23.120 | 39.125 | 6.585 |
| February 2004 | cold | S11 P-01 | 3.594 | 3.750 | 0.978 | 0.119 | 2.489 | 24 | 12.054 | 8.432 | 0.783 | 0.502 | 0.481 | 0.700 | 0.672 | 39.867 | 0.235 | 0.881 | 0.681 | 0.881 | 38.462 | 0.229 | 2.410 | 602 | 0.407 | 0.934 | 22.422 | 30.125 | 5.001 |
| March 2004 | cold | S11 P-01 | 2.803 | 2.936 | 0.519 | 0.130 | 2.356 | 22 | 10.547 | 7.666 | 0.762 | 0.479 | 0.455 | 0.727 | 0.698 | 12.263 | 0.245 | 0.870 | 0.654 | 0.870 | 43.590 | 0.224 | 2.326 | 1794 | 0.434 | 0.735 | 16.166 | 28.125 | 3.530 |
| April 2004 | cold | S11 P-01 | 3.772 | 3.898 | 0.581 | 0.128 | 2.447 | 31 | 11.556 | 7.815 | 0.713 | 0.373 | 0.352 | 0.676 | 0.646 | 10.904 | 0.295 | 0.872 | 0.654 | 0.872 | 20.513 | 0.243 | 2.421 | 2843 | 0.416 | 0.858 | 26.590 | 37.125 | 4.865 |
| May 2004 | warm | S11 P-01 | 3.122 | 3.252 | 0.535 | 0.124 | 2.383 | 25 | 10.837 | 8.034 | 0.740 | 0.433 | 0.410 | 0.741 | 0.715 | 11.468 | 0.267 | 0.876 | 0.661 | 0.876 | 35.897 | 0.194 | 2.356 | 2180 | 0.376 | 0.849 | 21.217 | 31.125 | 3.960 |
| June 2004 | warm | S11 P-01 | 4.472 | 4.616 | 1.000 | 0.086 | 2.726 | 32 | 15.275 | 11.587 | 0.787 | 0.477 | 0.460 | 0.759 | 0.742 | 31.220 | 0.227 | 0.914 | 0.727 | 0.914 | 17.949 | 0.138 | 2.662 | 1025 | 0.265 | 0.714 | 22.838 | 38.125 | 6.271 |
| March 2006 | cold | S11 P-02 | 2.396 | 2.522 | 0.379 | 0.455 | 1.497 | 20 | 4.468 | 2.197 | 0.500 | 0.223 | 0.183 | 0.492 | 0.345 | 7.186 | 0.510 | 0.545 | 0.331 | 0.545 | 48.718 | 0.667 | 1.479 | 2783 | 0.733 | 0.328 | 6.570 | 26.125 | 2.914 |
| June 2006 | warm | S11 P-02 | 2.520 | 2.653 | 0.461 | 0.124 | 2.378 | 20 | 10.786 | 8.034 | 0.794 | 0.539 | 0.515 | 0.745 | 0.719 | 10.633 | 0.211 | 0.876 | 0.662 | 0.876 | 48.718 | 0.210 | 2.351 | 1881 | 0.407 | 0.682 | 13.645 | 26.125 | 3.124 |
| September 2006 | warm | S11 P-02 | 2.903 | 3.049 | 0.670 | 0.137 | 2.335 | 21 | 10.332 | 7.276 | 0.767 | 0.492 | 0.467 | 0.704 | 0.673 | 21.407 | 0.244 | 0.863 | 0.649 | 0.863 | 46.154 | 0.253 | 2.288 | 981 | 0.452 | 0.980 | 20.578 | 27.125 | 3.774 |
| December 2006 | cold | S11 P-02 | 3.423 | 3.572 | 0.834 | 0.085 | 2.703 | 24 | 14.926 | 11.762 | 0.851 | 0.622 | 0.605 | 0.788 | 0.773 | 28.986 | 0.159 | 0.915 | 0.732 | 0.915 | 38.462 | 0.147 | 2.638 | 828 | 0.278 | 0.781 | 18.748 | 30.125 | 4.621 |
| September 2008 | warm | S11 P-02 | 3.988 | 4.147 | 1.132 | 0.114 | 2.490 | 26 | 12.065 | 8.748 | 0.764 | 0.464 | 0.443 | 0.725 | 0.700 | 49.242 | 0.259 | 0.886 | 0.689 | 0.886 | 33.333 | 0.205 | 2.400 | 528 | 0.377 | 0.800 | 20.803 | 32.125 | 5.735 |
| December 2008 | cold | S11 P-02 | 4.411 | 4.581 | 1.417 | 0.116 | 2.583 | 27 | 13.232 | 8.655 | 0.784 | 0.490 | 0.470 | 0.654 | 0.626 | 74.380 | 0.248 | 0.884 | 0.693 | 0.884 | 30.769 | 0.273 | 2.456 | 363 | 0.377 | 0.772 | 20.832 | 33.125 | 6.743 |
| March 2009 | cold | S11 P-02 | 5.053 | 5.215 | 1.489 | 0.078 | 2.817 | 32 | 16.729 | 12.853 | 0.813 | 0.523 | 0.507 | 0.768 | 0.754 | 69.264 | 0.212 | 0.922 | 0.753 | 0.922 | 17.949 | 0.154 | 2.697 | 462 | 0.281 | 0.747 | 23.920 | 38.125 | 7.811 |
| August 2009 | warm | S11 P-02 | 3.383 | 3.518 | 0.646 | 0.125 | 2.416 | 26 | 11.206 | 7.974 | 0.742 | 0.431 | 0.408 | 0.712 | 0.683 | 16.059 | 0.268 | 0.875 | 0.662 | 0.875 | 33.333 | 0.235 | 2.380 | 1619 | 0.389 | 0.877 | 22.800 | 32.125 | 4.399 |

**Table A.3**

Results of Shapiro’s, Bartett’s, Student’s t and Wilcoxon tests per index, when examining the tested samples’ ( S2 P-01 | S11 P-02 ) differentiation. A) all-year-round, B) warm-period-only and C) cold-period-only, data used. **In bold** the cases of statistically significant differentiation through Student’s t-test and/or Wilcoxon.

| **A** |  |  |  |  |  |
| --- | --- | --- | --- | --- | --- |
| Index | Shapiro for S2-P01 | Shapiro for S11-P02 | Bartlett’s | Student’s t-test | Wilcoxon |
| Margalef | 2.33E-02 | 7.42E-01 | 2.47E-01 |  | **1.23E-04** |
| Gleason | 1.44E-02 | 7.50E-01 | 2.66E-01 |  | **1.23E-04** |
| Menhinick | 9.16E-07 | 3.76E-01 | 6.16E-01 |  | **8.65E-04** |
| Simpson | 5.40E-02 | 8.24E-05 | 4.44E-02 |  | **5.98E-04** |
| Shannon | 1.33E-02 | 3.56E-02 | 1.64E-01 |  | **2.27E-04** |
| HillN0 | 4.51E-01 | 3.71E-01 | 5.82E-01 | **5.26E-09** | **5.05E-05** |
| HillN1 | 5.61E-05 | 5.67E-01 | 4.73E-01 |  | **2.27E-04** |
| HillN2 | 4.20E-06 | 3.21E-01 | 5.34E-01 |  | **5.98E-04** |
| E1 | 9.89E-02 | 4.05E-03 | 4.51E-02 |  | **1.48E-03** |
| E2 | 3.37E-03 | 1.51E-01 | 3.06E-01 |  | **6.51E-03** |
| E3 | 8.55E-04 | 1.16E-01 | 2.23E-01 |  | **3.45E-03** |
| E4 | 9.67E-02 | 4.86E-02 | 7.67E-01 | 3.27E-01 | 7.41E-01 |
| E5 | 4.17E-03 | 7.16E-03 | 5.59E-01 |  | **2.36E-02** |
| Odum | 2.12E-08 | 1.83E-01 | 1.75E-01 |  | **2.09E-03** |
| Redundancy | 9.61E-02 | 5.40E-03 | 4.40E-02 |  | **2.48E-03** |
| Pie | 5.40E-02 | 8.24E-05 | 4.44E-02 |  | **5.98E-04** |
| M | 6.54E-03 | 1.30E-03 | 1.83E-01 |  | **1.24E-03** |
| Tu | 5.40E-02 | 8.24E-05 | 4.44E-02 |  | **5.98E-04** |
| Kothe | 4.51E-01 | 3.71E-01 | 5.82E-01 | **5.26E-09** | **5.05E-05** |
| Berger-Parker | 2.58E-03 | 1.02E-03 | 2.76E-01 |  | **8.65E-04** |
| Brillouin | 1.80E-02 | 1.30E-02 | 1.53E-01 |  | **1.23E-04** |
| N | 4.74E-01 | 2.25E-01 | 9.73E-03 |  | **2.36E-02** |
| McNaughton | 2.64E-04 | 2.11E-02 | 3.21E-01 |  | **1.04E-03** |
| Camargo Evenness | 2.14E-04 | 9.42E-02 | 1.37E-01 |  | 5.65E-02 |
| Camargo Diversity | 2.78E-04 | 5.74E-02 | 6.33E-01 |  | **3.37E-04** |
| Chao 2 | 4.51E-01 | 3.71E-01 | 5.82E-01 | **5.26E-09** | **5.05E-05** |
| Fisher’s alpha | 1.63E-04 | 5.82E-01 | 1.30E-01 |  | **2.77E-04** |

| **B** |  |  |  |  |  |
| --- | --- | --- | --- | --- | --- |
| Index | Shapiro for S2-P01 | Shapiro for S11-P02 | Bartlett’s | Student’s t-test | Wilcoxon |
| Margalef | 4.03E-02 | 9.24E-01 | 8.22E-01 |  | **1.33E-02** |
| Gleason | 1.91E-02 | 9.28E-01 | 7.94E-01 |  | **1.33E-02** |
| Menhinick | 1.42E-06 | 3.61E-01 | 3.50E-01 |  | **2.84E-02** |
| Simpson | 1.39E-01 | 7.70E-01 | 1.18E-04 |  | **2.84E-02** |
| Shannon | 1.80E-01 | 9.11E-01 | 2.12E-03 |  | **1.96E-02** |
| HillN0 | 5.78E-01 | 9.96E-02 | 8.61E-01 | **9.94E-05** | **5.66E-03** |
| HillN1 | 3.08E-03 | 8.71E-01 | 2.57E-02 |  | **1.96E-02** |
| HillN2 | 1.26E-04 | 7.90E-01 | 1.53E-02 |  | **2.84E-02** |
| E1 | 3.90E-01 | 7.96E-01 | 1.77E-03 |  | **2.84E-02** |
| E2 | 5.92E-02 | 9.66E-01 | 2.86E-02 |  | **4.03E-02** |
| E3 | 1.86E-02 | 9.65E-01 | 1.95E-02 |  | **4.03E-02** |
| E4 | 1.65E-01 | 7.34E-01 | 1.27E-02 |  | 9.44E-01 |
| E5 | 3.28E-02 | 8.73E-01 | 2.70E-03 |  | 7.71E-02 |
| Odum | 3.26E-07 | 2.25E-01 | 4.92E-02 |  | **4.03E-02** |
| Redundancy | 3.92E-01 | 5.36E-01 | 2.81E-03 |  | **2.84E-02** |
| Pie | 1.39E-01 | 7.70E-01 | 1.18E-04 |  | **2.84E-02** |
| M | 6.47E-02 | 3.73E-01 | 1.03E-03 |  | **2.84E-02** |
| Tu | 1.39E-01 | 7.70E-01 | 1.18E-04 |  | **2.84E-02** |
| Kothe | 5.78E-01 | 9.96E-02 | 8.61E-01 | **9.94E-05** | **5.66E-03** |
| Berger-Parker | 4.21E-02 | 5.39E-01 | 1.51E-03 |  | **2.84E-02** |
| Brillouin | 2.30E-01 | 6.30E-01 | 1.15E-03 |  | **5.82E-03** |
| N | 1.93E-01 | 7.26E-01 | 5.98E-02 |  | 1.04E-01 |
| McNaughton | 3.33E-02 | 5.20E-01 | 8.03E-03 |  | **2.84E-02** |
| Camargo Evenness | 3.19E-03 | 9.96E-01 | 7.35E-02 |  | 4.37E-01 |
| Camargo Diversity | 1.28E-02 | 1.79E-01 | 5.64E-01 |  | **8.89E-03** |
| Chao 2 | 5.78E-01 | 9.96E-02 | 8.61E-01 | **9.94E-05** | **5.66E-03** |
| Fisher’s alpha | 3.23E-04 | 8.31E-01 | 7.13E-01 |  | **1.96E-02** |

| **C** |  |  |  |  |  |
| --- | --- | --- | --- | --- | --- |
| Index | Shapiro for S2-P01 | Shapiro for S11-P02 | Bartlett’s | Student’s t-test | Wilcoxon |
| Margalef | 1.63E-01 | 8.84E-01 | 1.73E-01 | **2.01E-04** | **8.89E-03** |
| Gleason | 1.71E-01 | 8.74E-01 | 1.77E-01 | **2.18E-04** | **8.89E-03** |
| Menhinick | 1.92E-02 | 4.24E-01 | 4.86E-02 |  | **1.33E-02** |
| Simpson | 2.01E-01 | 1.30E-02 | 5.31E-01 |  | **1.33E-02** |
| Shannon | 3.60E-02 | 6.01E-02 | 9.07E-01 |  | **1.33E-02** |
| HillN0 | 1.80E-01 | 9.89E-01 | 4.87E-01 | **5.08E-05** | **5.55E-03** |
| HillN1 | 3.30E-04 | 2.42E-01 | 1.34E-01 |  | **1.33E-02** |
| HillN2 | 5.01E-05 | 3.99E-01 | 7.55E-02 |  | **1.33E-02** |
| E1 | 2.04E-01 | 7.73E-02 | 5.77E-01 | **9.23E-03** | **1.96E-02** |
| E2 | 1.21E-02 | 4.17E-01 | 6.20E-01 |  | 7.71E-02 |
| E3 | 8.52E-03 | 3.77E-01 | 7.85E-01 |  | **4.03E-02** |
| E4 | 5.80E-01 | 3.97E-01 | 1.78E-01 | 2.83E-01 | 7.24E-01 |
| E5 | 1.55E-01 | 2.30E-01 | 4.32E-01 | 9.80E-02 | 2.29E-01 |
| Odum | 1.17E-03 | 3.88E-01 | 1.49E-02 |  | **2.84E-02** |
| Redundancy | 2.23E-01 | 1.84E-01 | 5.59E-01 | **9.65E-03** | **2.84E-02** |
| Pie | 2.01E-01 | 1.30E-02 | 5.31E-01 |  | **1.33E-02** |
| M | 2.97E-02 | 3.03E-02 | 9.99E-01 |  | **1.33E-02** |
| Tu | 2.01E-01 | 1.30E-02 | 5.31E-01 |  | **1.33E-02** |
| Kothe | 1.80E-01 | 9.89E-01 | 4.87E-01 | **5.08E-05** | **5.55E-03** |
| Berger-Parker | 1.63E-02 | 8.62E-02 | 9.15E-01 |  | **1.96E-02** |
| Brillouin | 4.20E-02 | 6.62E-02 | 8.40E-01 |  | **1.33E-02** |
| N | 7.00E-02 | 5.93E-02 | 1.45E-01 | 1.62E-01 | 7.71E-02 |
| McNaughton | 6.43E-04 | 6.68E-02 | 8.20E-01 |  | **1.96E-02** |
| Camargo Evenness | 4.19E-03 | 7.98E-03 | 9.20E-01 |  | 5.62E-02 |
| Camargo Diversity | 9.90E-04 | 2.92E-01 | 1.88E-01 |  | **1.33E-02** |
| Chao 2 | 1.80E-01 | 9.89E-01 | 4.87E-01 | **5.08E-05** | **5.55E-03** |
| Fisher’s alpha | 8.53E-02 | 8.04E-01 | 3.64E-02 |  | **1.33E-02** |

**Table A. 4**

All – year-round data. Classification of the remaining samplings in the two quality status groups, based on the LDA (indices used: “HillN0”, “Kothe”, “Chao 2”). In cases where posterior probability for “non-good” or “good status” was more than 0.95, the samplings’ status are presented **in** **bold**.

| Remaining  sampling | Station & Period | Non-good status  posterior probability | Good Status  posterior probability | LD function value | Status |
| --- | --- | --- | --- | --- | --- |
| 1 | **S2 P-02** | **1.00E+00** | **9.66E-06** | **-2.22E+00** | **Non-good status** |
| 2 | **S2 P-02** | **1.00E+00** | **1.39E-06** | **-2.76E+00** | **Non-good status** |
| 3 | **S2 P-02** | **9.97E-01** | **3.26E-03** | **-5.83E-01** | **Non-good status** |
| 4 | **S2 P-02** | **1.00E+00** | **1.39E-06** | **-2.76E+00** | **Non-good status** |
| 5 | **S7 P-01** | **3.82E-04** | **1.00E+00** | **3.23E+00** | **Good status** |
| 6 | **S7 P-01** | **1.82E-02** | **9.82E-01** | **2.14E+00** | **Good status** |
| 7 | S7 P-01 | 4.74E-01 | 5.26E-01 | 1.05E+00 | Good status |
| 8 | **S7 P-01** | **4.67E-02** | **9.53E-01** | **1.87E+00** | **Good status** |
| 9 | **S7 P-01** | **6.98E-03** | **9.93E-01** | **2.41E+00** | **Good status** |
| 10 | **S7 P-01** | **3.82E-04** | **1.00E+00** | **3.23E+00** | **Good status** |
| 11 | **S7 P-01** | **3.82E-04** | **1.00E+00** | **3.23E+00** | **Good status** |
| 12 | **S7 P-01** | **9.78E-01** | **2.23E-02** | **-3.89E-02** | **Non-good status** |
| 13 | **S7 P-01** | **1.82E-02** | **9.82E-01** | **2.14E+00** | **Good status** |
| 14 | **S7 P-01** | **1.01E-03** | **9.99E-01** | **2.96E+00** | **Good status** |
| 15 | **S7 P-01** | **5.48E-05** | **1.00E+00** | **3.77E+00** | **Good status** |
| 16 | **S7 P-01** | **7.86E-06** | **1.00E+00** | **4.32E+00** | **Good status** |
| 17 | **S7 P-01** | **2.65E-03** | **9.97E-01** | **2.68E+00** | **Good status** |
| 18 | **S7 P-01** | **4.67E-02** | **9.53E-01** | **1.87E+00** | **Good status** |
| 19 | **S7 P-01** | **5.48E-05** | **1.00E+00** | **3.77E+00** | **Good status** |
| 20 | **S7 P-01** | **6.98E-03** | **9.93E-01** | **2.41E+00** | **Good status** |
| 21 | **S7 P-01** | **3.82E-04** | **1.00E+00** | **3.23E+00** | **Good status** |
| 22 | **S7 P-01** | **2.07E-05** | **1.00E+00** | **4.04E+00** | **Good status** |
| 23 | **S7 P-01** | **4.67E-02** | **9.53E-01** | **1.87E+00** | **Good status** |
| 24 | S7 P-02 | 7.04E-01 | 2.96E-01 | 7.78E-01 | Non-good status |
| 25 | S7 P-02 | 9.43E-01 | 5.68E-02 | 2.33E-01 | Non-good status |
| 26 | S7 P-02 | 8.63E-01 | 1.37E-01 | 5.06E-01 | Non-good status |
| 27 | **S7 P-02** | **2.65E-03** | **9.97E-01** | **2.68E+00** | **Good status** |
| 28 | **S7 P-02** | **2.98E-06** | **1.00E+00** | **4.59E+00** | **Good status** |
| 29 | **S7 P-02** | **1.01E-03** | **9.99E-01** | **2.96E+00** | **Good status** |
| 30 | **S7 P-02** | **1.01E-03** | **9.99E-01** | **2.96E+00** | **Good status** |
| 31 | **S7 P-02** | **2.07E-05** | **1.00E+00** | **4.04E+00** | **Good status** |
| 32 | **S7 P-02** | **1.45E-04** | **1.00E+00** | **3.50E+00** | **Good status** |
| 33 | **S7 P-02** | **2.32E-08** | **1.00E+00** | **5.95E+00** | **Good status** |
| 34 | **S7 P-02** | **1.13E-06** | **1.00E+00** | **4.86E+00** | **Good status** |
| 35 | **S7 P-02** | **2.07E-05** | **1.00E+00** | **4.04E+00** | **Good status** |
| 36 | S11 P-0 | 4.74E-01 | 5.26E-01 | 1.05E+00 | Good status |
| 37 | **S11 P-0** | **3.82E-04** | **1.00E+00** | **3.23E+00** | **Good status** |
| 38 | S11 P-0 | 4.74E-01 | 5.26E-01 | 1.05E+00 | Good status |
| 39 | **S11 P-0** | **6.98E-03** | **9.93E-01** | **2.41E+00** | **Good status** |
| 40 | **S11 P-0** | **7.86E-06** | **1.00E+00** | **4.32E+00** | **Good status** |
| 41 | **S11 P-0** | **2.07E-05** | **1.00E+00** | **4.04E+00** | **Good status** |
| 42 | **S11 P-0** | **6.98E-03** | **9.93E-01** | **2.41E+00** | **Good status** |
| 43 | **S11 P-0** | **2.65E-03** | **9.97E-01** | **2.68E+00** | **Good status** |
| 44 | **S11 P-0** | **4.67E-02** | **9.53E-01** | **1.87E+00** | **Good status** |
| 45 | **S11 P-0** | **1.13E-06** | **1.00E+00** | **4.86E+00** | **Good status** |
| 46 | **S11 P-0** | **2.07E-05** | **1.00E+00** | **4.04E+00** | **Good status** |
| 47 | **S11 P-0** | **4.67E-02** | **9.53E-01** | **1.87E+00** | **Good status** |
| 48 | **S11 P-0** | **1.45E-04** | **1.00E+00** | **3.50E+00** | **Good status** |
| 49 | S11 P-0 | 8.63E-01 | 1.37E-01 | 5.06E-01 | Non-good status |
| 50 | S11 P-0 | 4.74E-01 | 5.26E-01 | 1.05E+00 | Good status |
| 51 | **S11 P-0** | **4.67E-02** | **9.53E-01** | **1.87E+00** | **Good status** |
| 52 | S11 P-0 | 2.55E-01 | 7.45E-01 | 1.32E+00 | Good status |
| 53 | **S11 P-0** | **9.99E-01** | **1.24E-03** | **-8.56E-01** | **Non-good status** |
| 54 | **S11 P-0** | **3.82E-04** | **1.00E+00** | **3.23E+00** | **Good status** |
| 55 | **S11 P-0** | **7.86E-06** | **1.00E+00** | **4.32E+00** | **Good status** |
| 56 | S11 P-0 | 2.55E-01 | 7.45E-01 | 1.32E+00 | Good status |
| 57 | **S11 P-0** | **5.48E-05** | **1.00E+00** | **3.77E+00** | **Good status** |
| 58 | **S11 P-01** | **2.65E-03** | **9.97E-01** | **2.68E+00** | **Good status** |
| 59 | **S11 P-01** | **4.67E-02** | **9.53E-01** | **1.87E+00** | **Good status** |
| 60 | **S11 P-01** | **1.01E-03** | **9.99E-01** | **2.96E+00** | **Good status** |
| 61 | **S11 P-01** | **7.86E-06** | **1.00E+00** | **4.32E+00** | **Good status** |
| 62 | **S11 P-01** | **5.48E-05** | **1.00E+00** | **3.77E+00** | **Good status** |
| 63 | **S11 P-01** | **1.82E-02** | **9.82E-01** | **2.14E+00** | **Good status** |
| 64 | **S11 P-01** | **1.82E-02** | **9.82E-01** | **2.14E+00** | **Good status** |
| 65 | **S11 P-01** | **5.48E-05** | **1.00E+00** | **3.77E+00** | **Good status** |
| 66 | **S11 P-01** | **3.82E-04** | **1.00E+00** | **3.23E+00** | **Good status** |
| 67 | **S11 P-01** | **5.48E-05** | **1.00E+00** | **3.77E+00** | **Good status** |
| 68 | **S11 P-01** | **2.65E-03** | **9.97E-01** | **2.68E+00** | **Good status** |
| 69 | **S11 P-01** | **1.45E-04** | **1.00E+00** | **3.50E+00** | **Good status** |
| 70 | **S11 P-01** | **7.86E-06** | **1.00E+00** | **4.32E+00** | **Good status** |
| 71 | **S11 P-01** | **2.65E-03** | **9.97E-01** | **2.68E+00** | **Good status** |
| 72 | **S11 P-01** | **6.98E-03** | **9.93E-01** | **2.41E+00** | **Good status** |
| 73 | **S11 P-01** | **3.82E-04** | **1.00E+00** | **3.23E+00** | **Good status** |
| 74 | S11 P-01 | 2.55E-01 | 7.45E-01 | 1.32E+00 | Good status |
| 75 | **S11 P-01** | **3.82E-04** | **1.00E+00** | **3.23E+00** | **Good status** |
| 76 | **S11 P-01** | **1.82E-02** | **9.82E-01** | **2.14E+00** | **Good status** |
| 77 | **S11 P-01** | **6.98E-03** | **9.93E-01** | **2.41E+00** | **Good status** |
| 78 | **S11 P-01** | **1.82E-02** | **9.82E-01** | **2.14E+00** | **Good status** |
| 79 | **S11 P-01** | **5.48E-05** | **1.00E+00** | **3.77E+00** | **Good status** |
| 80 | **S11 P-01** | **3.82E-04** | **1.00E+00** | **3.23E+00** | **Good status** |
| 81 | **S11 P-01** | **7.86E-06** | **1.00E+00** | **4.32E+00** | **Good status** |
| 82 | **S11 P-01** | **1.45E-04** | **1.00E+00** | **3.50E+00** | **Good status** |
| 83 | **S11 P-01** | **3.82E-04** | **1.00E+00** | **3.23E+00** | **Good status** |
| 84 | **S11 P-01** | **1.82E-02** | **9.82E-01** | **2.14E+00** | **Good status** |
| 85 | **S11 P-01** | **6.98E-03** | **9.93E-01** | **2.41E+00** | **Good status** |
| 86 | **S11 P-01** | **2.65E-03** | **9.97E-01** | **2.68E+00** | **Good status** |
| 87 | S11 P-01 | 1.15E-01 | 8.85E-01 | 1.59E+00 | Good status |
| 88 | **S11 P-01** | **1.45E-04** | **1.00E+00** | **3.50E+00** | **Good status** |
| 89 | **S11 P-01** | **1.13E-06** | **1.00E+00** | **4.86E+00** | **Good status** |
| 90 | **S11 P-01** | **1.01E-03** | **9.99E-01** | **2.96E+00** | **Good status** |
| 91 | **S11 P-01** | **2.98E-06** | **1.00E+00** | **4.59E+00** | **Good status** |
| 92 | **S11 P-01** | **6.98E-03** | **9.93E-01** | **2.41E+00** | **Good status** |
| 93 | **S11 P-01** | **7.86E-06** | **1.00E+00** | **4.32E+00** | **Good status** |
| 94 | **S11 P-01** | **1.62E-07** | **1.00E+00** | **5.41E+00** | **Good status** |
| 95 | **S11 P-01** | **1.45E-04** | **1.00E+00** | **3.50E+00** | **Good status** |
| 96 | **S11 P-01** | **5.48E-05** | **1.00E+00** | **3.77E+00** | **Good status** |
| 97 | **S11 P-01** | **7.86E-06** | **1.00E+00** | **4.32E+00** | **Good status** |
| 98 | **S11 P-01** | **2.98E-06** | **1.00E+00** | **4.59E+00** | **Good status** |
| 99 | **S11 P-01** | **4.27E-07** | **1.00E+00** | **5.13E+00** | **Good status** |
| 100 | **S11 P-01** | **2.07E-05** | **1.00E+00** | **4.04E+00** | **Good status** |
| 101 | **S11 P-01** | **1.01E-03** | **9.99E-01** | **2.96E+00** | **Good status** |
| 102 | **S11 P-01** | **1.45E-04** | **1.00E+00** | **3.50E+00** | **Good status** |
| 103 | **S11 P-01** | **1.45E-04** | **1.00E+00** | **3.50E+00** | **Good status** |
| 104 | **S11 P-01** | **6.12E-08** | **1.00E+00** | **5.68E+00** | **Good status** |
| 105 | **S11 P-01** | **6.12E-08** | **1.00E+00** | **5.68E+00** | **Good status** |
| 106 | **S11 P-01** | **1.13E-06** | **1.00E+00** | **4.86E+00** | **Good status** |
| 107 | **S11 P-01** | **6.98E-03** | **9.93E-01** | **2.41E+00** | **Good status** |
| 108 | **S11 P-01** | **4.67E-02** | **9.53E-01** | **1.87E+00** | **Good status** |
| 109 | **S11 P-01** | **7.86E-06** | **1.00E+00** | **4.32E+00** | **Good status** |
| 110 | **S11 P-01** | **2.65E-03** | **9.97E-01** | **2.68E+00** | **Good status** |
| 111 | **S11 P-01** | **2.98E-06** | **1.00E+00** | **4.59E+00** | **Good status** |

**Table A. 5**

Summer data. Classification of the remaining samplings in the two quality status groups, based on the LDA (indices used: “HillN0”, “Kothe”, “Chao 2”). In cases where posterior probability for “non-good” or “good status” was more than 0.95, the samplings’ status are presented **in bold**.

| Remaining sampling | Station & Period | Non-good status posterior probability | Good Status posterior probability | LD function value | Status |
| --- | --- | --- | --- | --- | --- |
| 1 | **S2 P-02** | **9.95E-01** | **4.87E-03** | **-5.84E-01** | **Non-good status** |
| 2 | **S2 P-02** | **1.00E+00** | **1.87E-06** | **-2.92E+00** | **Non-good status** |
| 3 | S7 P-01 | 3.58E-01 | 6.42E-01 | 1.17E+00 | Good status |
| 4 | **S7 P-01** | **2.83E-02** | **9.72E-01** | **2.04E+00** | **Good status** |
| 5 | **S7 P-01** | **4.05E-03** | **9.96E-01** | **2.63E+00** | **Good status** |
| 6 | **S7 P-01** | **9.66E-01** | **3.38E-02** | **8.13E-08** | **Non-good status** |
| 7 | **S7 P-01** | **1.08E-02** | **9.89E-01** | **2.34E+00** | **Good status** |
| 8 | **S7 P-01** | **4.15E-06** | **1.00E+00** | **4.67E+00** | **Good status** |
| 9 | **S7 P-01** | **1.52E-03** | **9.98E-01** | **2.92E+00** | **Good status** |
| 10 | **S7 P-01** | **4.05E-03** | **9.96E-01** | **2.63E+00** | **Good status** |
| 11 | **S7 P-01** | **2.12E-04** | **1.00E+00** | **3.50E+00** | **Good status** |
| 12 | S7 P-02 | 9.14E-01 | 8.57E-02 | 2.92E-01 | Non-good status |
| 13 | S7 P-02 | 8.00E-01 | 2.00E-01 | 5.84E-01 | Non-good status |
| 14 | **S7 P-02** | **5.68E-04** | **9.99E-01** | **3.21E+00** | **Good status** |
| 15 | **S7 P-02** | **5.68E-04** | **9.99E-01** | **3.21E+00** | **Good status** |
| 16 | **S7 P-02** | **7.94E-05** | **1.00E+00** | **3.79E+00** | **Good status** |
| 17 | **S7 P-02** | **1.11E-05** | **1.00E+00** | **4.38E+00** | **Good status** |
| 18 | **S11 P-0** | **2.12E-04** | **1.00E+00** | **3.50E+00** | **Good status** |
| 19 | S11 P-0 | 3.58E-01 | 6.42E-01 | 1.17E+00 | Good status |
| 20 | **S11 P-0** | **4.05E-03** | **9.96E-01** | **2.63E+00** | **Good status** |
| 21 | **S11 P-0** | **1.52E-03** | **9.98E-01** | **2.92E+00** | **Good status** |
| 22 | **S11 P-0** | **2.83E-02** | **9.72E-01** | **2.04E+00** | **Good status** |
| 23 | S11 P-0 | 8.00E-01 | 2.00E-01 | 5.84E-01 | Non-good status |
| 24 | S11 P-0 | 3.58E-01 | 6.42E-01 | 1.17E+00 | Good status |
| 25 | **S11 P-0** | **9.98E-01** | **1.83E-03** | **-8.76E-01** | **Non-good status** |
| 26 | S11 P-0 | 1.72E-01 | 8.28E-01 | 1.46E+00 | Good status |
| 27 | **S11 P-0** | **2.97E-05** | **1.00E+00** | **4.09E+00** | **Good status** |
| 28 | **S11 P-01** | **2.83E-02** | **9.72E-01** | **2.04E+00** | **Good status** |
| 29 | **S11 P-01** | **2.12E-04** | **1.00E+00** | **3.50E+00** | **Good status** |
| 30 | **S11 P-01** | **4.05E-03** | **9.96E-01** | **2.63E+00** | **Good status** |
| 31 | **S11 P-01** | **2.12E-04** | **1.00E+00** | **3.50E+00** | **Good status** |
| 32 | S11 P-01 | 1.72E-01 | 8.28E-01 | 1.46E+00 | Good status |
| 33 | **S11 P-01** | **4.05E-03** | **9.96E-01** | **2.63E+00** | **Good status** |
| 34 | **S11 P-01** | **1.08E-02** | **9.89E-01** | **2.34E+00** | **Good status** |
| 35 | **S11 P-01** | **2.97E-05** | **1.00E+00** | **4.09E+00** | **Good status** |
| 36 | **S11 P-01** | **2.12E-04** | **1.00E+00** | **3.50E+00** | **Good status** |
| 37 | **S11 P-01** | **4.05E-03** | **9.96E-01** | **2.63E+00** | **Good status** |
| 38 | **S11 P-01** | **1.52E-03** | **9.98E-01** | **2.92E+00** | **Good status** |
| 39 | S11 P-01 | 7.23E-02 | 9.28E-01 | 1.75E+00 | Good status |
| 40 | **S11 P-01** | **7.94E-05** | **1.00E+00** | **3.79E+00** | **Good status** |
| 41 | **S11 P-01** | **4.15E-06** | **1.00E+00** | **4.67E+00** | **Good status** |
| 42 | **S11 P-01** | **8.10E-08** | **1.00E+00** | **5.84E+00** | **Good status** |
| 43 | **S11 P-01** | **7.94E-05** | **1.00E+00** | **3.79E+00** | **Good status** |
| 44 | **S11 P-01** | **2.97E-05** | **1.00E+00** | **4.09E+00** | **Good status** |
| 45 | **S11 P-01** | **5.68E-04** | **9.99E-01** | **3.21E+00** | **Good status** |
| 46 | **S11 P-01** | **7.94E-05** | **1.00E+00** | **3.79E+00** | **Good status** |
| 47 | **S11 P-01** | **7.94E-05** | **1.00E+00** | **3.79E+00** | **Good status** |
| 48 | **S11 P-01** | **3.03E-08** | **1.00E+00** | **6.13E+00** | **Good status** |
| 49 | **S11 P-01** | **1.52E-03** | **9.98E-01** | **2.92E+00** | **Good status** |
| 50 | **S11 P-01** | **1.55E-06** | **1.00E+00** | **4.96E+00** | **Good status** |

**Table A. 6**

Winter data. Classification of the remaining samplings in the two quality status groups, based on the LDA (indices used: “HillN0”, “Kothe”, “Chao 2”). In cases where posterior probability for “non-good” or “good status” was more than 0.95, the samplings’ status are presented **in bold**.

| Remaining sampling | Station & Period | Non-good status posterior probability | Good Status posterior probability | LD function value | Status |
| --- | --- | --- | --- | --- | --- |
| 1 | **S2 P-02** | **0.9999861** | **1.39203E-05** | **-2.052016** | **Non-good status** |
| 2 | **S2 P-02** | **0.9999977** | **2.30775E-06** | **-2.54733** | **Non-good status** |
| 3 | **S7 P-01** | **0.001125144** | **0.9988749** | **2.901126** | **Good status** |
| 4 | **S7 P-01** | **0.03937151** | **0.9606285** | **1.910497** | **Good status** |
| 5 | **S7 P-01** | **0.001125144** | **0.9988749** | **2.901126** | **Good status** |
| 6 | **S7 P-01** | **0.001125144** | **0.9988749** | **2.901126** | **Good status** |
| 7 | **S7 P-01** | **0.002758859** | **0.9972411** | **2.653469** | **Good status** |
| 8 | **S7 P-01** | **0.000186703** | **0.9998133** | **3.396439** | **Good status** |
| 9 | S7 P-01 | 0.0914546 | 0.9085454 | 1.66284 | Good status |
| 10 | **S7 P-01** | **0.000186703** | **0.9998133** | **3.396439** | **Good status** |
| 11 | **S7 P-01** | **7.602678E-05** | **0.999924** | **3.644097** | **Good status** |
| 12 | S7 P-01 | 0.0914546 | 0.9085454 | 1.66284 | Good status |
| 13 | S7 P-02 | 0.785527 | 0.214473 | 0.6722121 | Non-good status |
| 14 | **S7 P-02** | **0.006748716** | **0.9932513** | **2.405811** | **Good status** |
| 15 | **S7 P-02** | **1.260461E-05** | **0.9999874** | **4.139411** | **Good status** |
| 16 | **S7 P-02** | **7.602678E-05** | **0.999924** | **3.644097** | **Good status** |
| 17 | **S7 P-02** | **1.410499E-07** | **0.9999999** | **5.377696** | **Good status** |
| 18 | **S7 P-02** | **5.132164E-06** | **0.9999949** | **4.387068** | **Good status** |
| 19 | S11 P-0 | 0.5985983 | 0.4014017 | 0.9198691 | Non-good status |
| 20 | **S11 P-0** | **3.095664E-05** | **0.999969** | **3.891754** | **Good status** |
| 21 | **S11 P-0** | **7.602678E-05** | **0.999924** | **3.644097** | **Good status** |
| 22 | **S11 P-0** | **0.01641371** | **0.9835863** | **2.158154** | **Good status** |
| 23 | **S11 P-0** | **5.132164E-06** | **0.9999949** | **4.387068** | **Good status** |
| 24 | **S11 P-0** | **7.602678E-05** | **0.999924** | **3.644097** | **Good status** |
| 25 | S11 P-0 | 0.0914546 | 0.9085454 | 1.66284 | Good status |
| 26 | **S11 P-0** | **0.0004584221** | **0.9995416** | **3.148783** | **Good status** |
| 27 | S11 P-0 | 0.0914546 | 0.9085454 | 1.66284 | Good status |
| 28 | S11 P-0 | 0.3777957 | 0.6222043 | 1.167526 | Good status |
| 29 | **S11 P-0** | **0.001125144** | **0.9988749** | **2.901126** | **Good status** |
| 30 | **S11 P-0** | **3.095664E-05** | **0.999969** | **3.891754** | **Good status** |
| 31 | **S11 P-01** | **0.006748716** | **0.9932513** | **2.405811** | **Good status** |
| 32 | **S11 P-01** | **0.002758859** | **0.9972411** | **2.653469** | **Good status** |
| 33 | **S11 P-01** | **3.095664E-05** | **0.999969** | **3.891754** | **Good status** |
| 34 | **S11 P-01** | **0.000186703** | **0.9998133** | **3.396439** | **Good status** |
| 35 | **S11 P-01** | **0.03937151** | **0.9606285** | **1.910497** | **Good status** |
| 36 | **S11 P-01** | **0.03937151** | **0.9606285** | **1.910497** | **Good status** |
| 37 | **S11 P-01** | **0.000186703** | **0.9998133** | **3.396439** | **Good status** |
| 38 | **S11 P-01** | **0.000186703** | **0.9998133** | **3.396439** | **Good status** |
| 39 | **S11 P-01** | **0.006748716** | **0.9932513** | **2.405811** | **Good status** |
| 40 | **S11 P-01** | **0.0004584221** | **0.9995416** | **3.148783** | **Good status** |
| 41 | **S11 P-01** | **3.095664E-05** | **0.999969** | **3.891754** | **Good status** |
| 42 | **S11 P-01** | **0.006748716** | **0.9932513** | **2.405811** | **Good status** |
| 43 | **S11 P-01** | **0.001125144** | **0.9988749** | **2.901126** | **Good status** |
| 44 | **S11 P-01** | **0.03937151** | **0.9606285** | **1.910497** | **Good status** |
| 45 | **S11 P-01** | **3.095664E-05** | **0.999969** | **3.891754** | **Good status** |
| 46 | **S11 P-01** | **0.0004584221** | **0.9995416** | **3.148783** | **Good status** |
| 47 | **S11 P-01** | **0.001125144** | **0.9988749** | **2.901126** | **Good status** |
| 48 | **S11 P-01** | **0.03937151** | **0.9606285** | **1.910497** | **Good status** |
| 49 | **S11 P-01** | **5.132164E-06** | **0.9999949** | **4.387068** | **Good status** |
| 50 | **S11 P-01** | **0.002758859** | **0.9972411** | **2.653469** | **Good status** |
| 51 | **S11 P-01** | **1.260461E-05** | **0.9999874** | **4.139411** | **Good status** |
| 52 | **S11 P-01** | **0.01641371** | **0.9835863** | **2.158154** | **Good status** |
| 53 | **S11 P-01** | **3.095664E-05** | **0.999969** | **3.891754** | **Good status** |
| 54 | **S11 P-01** | **1.260461E-05** | **0.9999874** | **4.139411** | **Good status** |
| 55 | **S11 P-01** | **2.089628E-06** | **0.9999979** | **4.634725** | **Good status** |
| 56 | **S11 P-01** | **7.602678E-05** | **0.999924** | **3.644097** | **Good status** |
| 57 | **S11 P-01** | **3.464217E-07** | **0.9999997** | **5.130039** | **Good status** |
| 58 | **S11 P-01** | **5.132164E-06** | **0.9999949** | **4.387068** | **Good status** |
| 59 | **S11 P-01** | **0.01641371** | **0.9835863** | **2.158154** | **Good status** |
| 60 | S11 P-01 | 0.0914546 | 0.9085454 | 1.66284 | Good status |
| 61 | **S11 P-01** | **3.095664E-05** | **0.999969** | **3.891754** | **Good status** |

**References**

Berger, W. H. and Parker, F. L. (1970) Diversity of Planktonic Foraminifera in Deep-Sea Sediments. *Science (80-. ).*, **168**, 1345–1347.

Camargo, J. (1992) New diversity index for assessing structural alterations in aquatic communities. *Bull. Environ. Contam. Toxicol.*, **48**, 428–434.

Chao, A. (2006) Species Estimation and Applications. *Encyclopedia of Statistical Sciences*. John Wiley & Sons, Inc., Hoboken, NJ, USA.

Fisher, R. A., Corbet, A. S., and Williams, C. B. (1943) The Relation Between the Number of Species and the Number of Individuals in a Random Sample of an Animal Population.

Hurlbert, S. H. (1971) The Nonconcept of Species Diversity: A Critique and Alternative Parameters. *Ecology*, **52**, 577–586.

Keefe, T. J. and Bergersen, E. P. (1977) A simple diversity index based on the theory of runs. *Water Res.*, **11**, 689–691.

Ludwig, J. A. and Reynolds, J. F. (1988) *Statistical ecology : a primer on methods and computing*. John Wiley & Sons, Ltd, New York :

Margalef, R. (1958) Information theory in ecology. *Gen. Syst.*, **3**, 36–71.

McIntosh, R. P. (1967) An Index of Diversity and the Relation of Certain Concepts to Diversity. *Ecology*, **48**, 392–404.

McNaughton, S. J. (1967) Relationships among Functional Properties of Californian Grassland. *Nature*, **216**, 168–169.

Menhinick, E. F. (1964) A Comparison of Some Species-Individuals Diversity Indices Applied to Samples of Field Insects.

Odum, H. T., Cantlon, J. E., and Kornicker, L. S. (1960) An Organizational Hierarchy Postulate for the Interpretation of Species-Individual Distributions, Species Entropy, Ecosystem Evolution, and the Meaning of a Species-Variety Index. *Ecology*, **41**, 395–395.

Patten, B. C. (1962) IMPROVED METHOD FOR ESTIMATING STABILITY IN PLANKTON1. *Limnol. Oceanogr.*, **7**, 266–268.

Pielou, E. C. (1975) *Ecological diversity*. John Wiley & Sons, Ltd.

Sheldon, A. L. (1969) Equitability Indices: Dependence on the Species Count.
